# Gpr116 Receptor Regulates Distinctive Functions in Pneumocytes and Vascular Endothelium

**DOI:** 10.1371/journal.pone.0137949

**Published:** 2015-09-22

**Authors:** Colin Niaudet, Jennifer J. Hofmann, Maarja A. Mäe, Bongnam Jung, Konstantin Gaengel, Michael Vanlandewijck, Elisabet Ekvärn, M. Dolores Salvado, Annika Mehlem, Sahar Al Sayegh, Liqun He, Thibaud Lebouvier, Marco Castro-Freire, Kan Katayama, Kjell Hultenby, Christine Moessinger, Philip Tannenberg, Sara Cunha, Kristian Pietras, Bàrbara Laviña, JongWook Hong, Tove Berg, Christer Betsholtz

**Affiliations:** 1 Department of Immunology, Genetics and Pathology, Rudbeck Laboratory, Uppsala University, Uppsala, Sweden; 2 Division of Vascular Biology, Department of Medical Biochemistry and Biophysics, Karolinska Institute, Stockholm, Sweden; 3 Physiological Chemistry II, Department of Medical Biochemistry and Biophysics, Karolinska Institute, Stockholm, Sweden; 4 Department of Laboratory Medicine, Division of Clinical Research Center, and Karolinska Institute, Stockholm, Sweden; 5 Department of Molecular Medicine and Surgery, Division of Vascular Surgery, Karolinska Institute, Stockholm, Sweden; 6 Lund University, Department of Laboratory Medicine, Medicon Village, Lund, Sweden; The Hospital for Sick Children and The University of Toronto, CANADA

## Abstract

Despite its known expression in both the vascular endothelium and the lung epithelium, until recently the physiological role of the adhesion receptor Gpr116/ADGRF5 has remained elusive. We generated a new mouse model of constitutive Gpr116 inactivation, with a large genetic deletion encompassing exon 4 to exon 21 of the *Gpr116* gene. This model allowed us to confirm recent results defining Gpr116 as necessary regulator of surfactant homeostasis. The loss of Gpr116 provokes an early accumulation of surfactant in the lungs, followed by a massive infiltration of macrophages, and eventually progresses into an emphysema-like pathology. Further analysis of this knockout model revealed cerebral vascular leakage, beginning at around 1.5 months of age. Additionally, endothelial-specific deletion of Gpr116 resulted in a significant increase of the brain vascular leakage. Mice devoid of Gpr116 developed an anatomically normal and largely functional vascular network, surprisingly exhibited an attenuated pathological retinal vascular response in a model of oxygen-induced retinopathy. These data suggest that Gpr116 modulates endothelial properties, a previously unappreciated function despite the pan-vascular expression of this receptor. Our results support the key pulmonary function of Gpr116 and describe a new role in the central nervous system vasculature.

## Introduction

The adhesion receptors are the most recently described of the 5 classes of seven transmembrane receptors. In mammals, each of the 33 members of this class is characterized by the presence of long N-terminal domains harboring various adhesion motifs and a cleavable so-called GPS domain [1]. To date, specific *in vivo* functions have been assigned to only a handful of the adhesions receptors. In a few cases, targeted loss-of-function mutations have revealed critical roles in key biological processes.

So far, the three Cadherin-EGF LAG Seven-pass G-type Receptor (Celsr, now ADGRC [2]) genes have been the most extensively studied of the adhesion receptors. Various experimental approaches ranging from mutagenesis to pharmacological modulation in organ explants show that Celsr proteins control planar cell polarity in several developmental processes, including neural tube closure [3], hair follicle formation [4] and lung morphogenesis [5] (for Celsr1/ADGRC1), ciliogenesis (for Celsr2/ADGRC2) [6], pancreas formation [7] and axon guidance [8] (for Celsr3/ADGRC3).

In the nervous system, three different adhesion receptors have been shown to take part in essential developmental processes. Gpr126/ADGRG6 is required for Schwann cell maturation and nerve myelination [9], a role conserved between zebrafish and mouse [10]. The involvement of two heterotrimeric G proteins downstream of Gpr126 have also been demonstrated [11]. Gpr56/ADGRG1 shapes the perisylvian gyri through control of neocortex progenitors [12]. Mutations in the human gene, *GPR56*, cause a malformation of the cerebral cortex known as bilateral frontoparietal polymicrogyria [13]. Finally, Gpr124/ADGRA2 has proven essential for angiogenesis in the neural tube and forebrain, and its global or specific endothelial deletion lead to hemorrhagic glomeruloid vascular malformations in the developing central nervous system (CNS) [14][15][16].

Pioneering work identified Gpr116, recently renamed ADGRF5 [2], as a new adhesion receptor expressed in the alveolar wall of rat lung [17]. Gpr116 was subsequently recognized as part of cluster VI, a group of 5 related receptors [1] whose tissue expression patterns have recently been mapped [18]. During late gestational stages, expression of Gpr116 was shown to increase in the lungs, suggesting a role in organogenesis [19][20]. Recently, three independent models of knockout mice for *Gpr116* have been used to investigate its function in the lung [19][20][21]. The absence of this gene leads to increased levels of saturated phosphatidylcholine (Sat PC), an essential component of surfactant, from 1 week of age onward [20]. This phenotype was also confirmed in older knockouts and was described as part of a global increase in all surfactant phospholipids at 4 weeks of age [19], accompanied by a shift in their carbon saturation at 8 weeks [21]. Surfactant proteins were also increased in *Gpr116* knockout mice [19][20][21]. The increased surfactant production was paralleled by expansion of alveolar macrophages, starting from 3 and peaking at 12 weeks of age [20]. In adult mice, this expansion developed into a severe pulmonary phenotype, with massive increases in surfactant phospholipids and proteins, as well as hyperplasia and hypertrophy of lipid-laden macrophages [19][20][21]. Importantly, the lung defect of the full *Gpr116* knockout could be reproduced by cell type-specific *Gpr116* knockout in type II pneumocytes, suggesting that Gpr116 plays a direct role in the surfactant producing cells [21]. However, this role is not yet completely understood.

We previously identified *Gpr116* as part of a cluster of 58 genes specifically expressed in blood vessel endothelium. Besides known classical endothelial markers, this cluster contained a handful of new, but poorly characterized GPCRs, one of which was *Gpr116* [22]. Other data supported the expression of Gpr116 in endothelial cells in early mouse embryos [23], as well as in normal adult and tumor tissues [21][20][24], and in circulating endothelial cells [25]. Its endothelial expression, however, remains debated, especially in the lungs[19][21]. Additionally, up until this point, the only reported defect linked to the loss of Gpr116 is lung pathology caused by surfactant overproduction [21] and a possible adipose tissue deregulation [26]. However, whether Gpr116 exerts a functional role in the vascular endothelium is still unknown.

The CNS endothelial cells acquire a high level of specialization [27]. Continuous complexes of tight junctions and reduced transcellular vesicular transport are the key features of the endothelium establishing the blood-brain-barrier (BBB) at embryonic day 15.5 [28]. Opening of the BBB represents a critical step in drug delivery to the brain. On the other hand, BBB disruption also plays a pivotal role in many neurological disorders [29]. It is frequently accompanied by cellular hypertrophy of the astrocytes and an increase in their intermediate-filament glial fibrillary acidic protein (GFAP) expression, a hallmark globally referred to as “reactive astrogliosis” [30][31][32].

The retina became a powerful *in vivo* model to study vascular patterning of the CNS vasculature in both developmental and pathological situations [33][34]. The retinal vasculature develops postnatally, relying on sprouting angiogenesis. In mice, the primary vascular plexus expands progressively to reach the entire retina by postnatal day (P) 7 with a structured network of arteries, veins and capillaries. In a broad range of retinopathies, vascular tufts represent the final stage, and can be modeled in mice by a short challenge of one-week-old pups to hyperoxia, leading to vaso-obliteration. Furthermore, upon return to normoxia, the vasculature will form neovascular tufts extending toward the vitreous. The model of “oxygen-induced retinopathy” (OIR) allows for quick assessment of the involvement of new pathways in the modulation of proliferative vascular disease [33][34].

In the present study, we report on a new murine model of *Gpr116* gene inactivation, in which a large deletion was generated, encompassing the predicted key functional domains of the Gpr116 receptor. Phenotypic analysis of these mice confirmed the severe deregulation of pulmonary surfactant in the absence of Gpr116. This alteration of surfactant quality temporally precedes a massive accumulation of foamy macrophages. In addition to this lung phenotype, our data also suggest a role for Gpr116 in the vascular endothelium *in vivo*, since *Gpr116* knockout mice display increased vascular leakage, and a normalized vascular response in the OIR model.

## Materials and Methods

### Ethics statement

Animal housing, as well as the experiments performed, were in accordance with Swedish legislation and were approved by the local animal ethics committees prior to experimentation. The protocols included in this study were approved by the Stockholm’s North Committee on the Ethics of Animal Experiments (permit numbers N33/10 and N15/12) and by the Uppsala Committee (permit number C224/12). All efforts were made to minimize animal suffering, and all surgery procedures were performed under anesthesia (Ketamin (75mg/kg) and Dexdomitor (0.5mg/kg)).

### Generation of *Gpr116* knockout mice and genotyping

The *Gpr116* ^−/−^ Velocigene mouse line was generated by Regeneron using a VelociGene approach[35]. 33 heterozygous mice were generated, of which 7 breeding pairs were used to generate the *Gpr116* knockout colony. The experiments were performed using mice with a mixed 129S6Sv/C57BL6 background. A part of the colony was simultaneously backcrossed to C57BL6 mice, for at least 6 generations. The backcrossed mice demonstrated no differences with the phenotypes described in this report.

**Fig 1:**
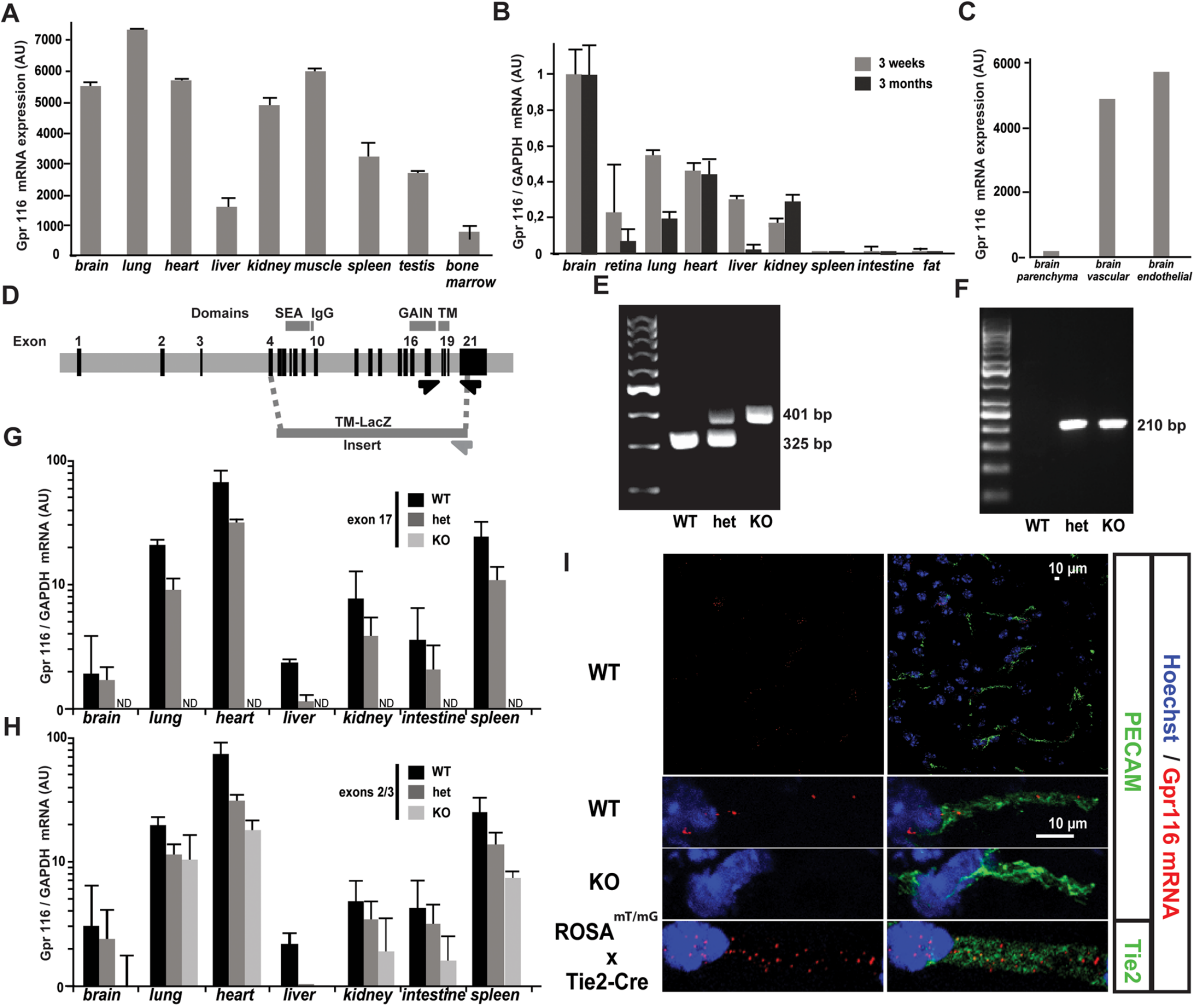
Vascular expression and genetic ablation of the *Gpr116* gene in mouse. A. *Gpr116* mRNA expression in the published organ-specific EC mRNA dataset [45]. B. *Gpr116* mRNA expression assessed by qRT-PCR in EC from 3-weeks-old and 3-months-old ROSA^mT/mG^ x Tie2-Cre mice. Results are normalized by brain EC expression. Error bars represent SD. (n = 3 mice per genotype). C. *Gpr116* mRNA expression in the published brain-specific vascular and EC mRNA dataset [46]. D. Schematic representation of the area targeted by homologous recombination in the *Gpr116* locus. Dotted lines indicate the regions of homology in between the Gpr116 locus and the cassette. The dark grey arrow indicates the position of WT primers: both are located in the untranslated region of exon 21, but the area recognized by the forward primer is lost in the mutant allele. The light grey arrow represents the knockout primer, specific for the cassette. Critical Gpr116 domains (SEA, IgG, GAIN and transmembraine, TM) are indicated above the corresponding encoding exons. E. Example of genotyping PCR products on genomic DNA (toe) from *Gpr116* WT, heterozygous and knockout littermates. WT primers amplify a 325-bp fragment in the 3´UTR exon 21 of *Gpr116* gene representing the wild type allele. The 401 bp band is specific for the mutant allele. F. Example of genotyping PCR products using genomic DNA (toe) from *Gpr116* WT, heterozygous and knockout littermates. LacZ primers amplify a 210 bp fragment in LacZ gene present in the insert replacing exon 4 to 21. G. *Gpr116* exon 17–18 mRNA expression assessed by qRT-PCR in *Gpr116* WT, heterozygous and knockout organs at P4 (n = 3 mice per genotype). H. *Gpr116* exon 2–3 mRNA expression assessed by qRT-PCR in *Gpr116* WT, heterozygous and knockout organs at P4 (n = 3 mice per genotype). I. mRNA detection by RNAscope in brain cortical capillary vessels from *Gpr116* WT (top row), knockout (middle row) and ROSA^mTmG^ X Tie2-Cre mice (lower row) at 3 weeks. On the left column, note that only the probe signal (red) and the nuclear staining (blue) are visible. On the right column, an endothelial staining (green) is merged to the probe and the nuclear signal: a CD31 antibody staining is on the two upper rows, while Tie-2 Cre mediated GFP is on the lower row. (n = 1 mouse per genotype).

Knockout and heterozygous pups were identified through genotyping PCR (Fig 1E) using the following primers: forward: 5´-GGGTAACGTGCTCTCTCTGC-3´, reverse wildtype: 5´-TGAACTCCTGGATACTAGCC-3´ or reverse knockout: 5´-TCATTCTCAGTATTGTTTTGCC-3´). The predicted amplicon sizes are 325 base pairs (bp) for the *Gpr116* wild type band, and 401 bp for the knockout allele.

For amplification of the Lacz reporter gene, the following primers were used: forward 5'-GGTAAACTGGCTCGGATTAGGG-3' and reverse: 5'-TTGACTGTAGCGGCTGATGTTG-3'. The predicted amplicon size is 210 bp.

### *Gpr116* AEC and EC knockout generation

Gpr116^Δexon17^ mice, generated by Novartis [**19**], were crossed to mice expressing either constitutive Sftpc-Cre [**36**], or the inducible VE-cad:Cre ER [**37**]. Offsprings were intercrossed to generate mice homozygotes for the floxed *Gpr116* allele, and carrying one copy of either Sftpc-Cre or VE-cad:Cre ER, referred as “*Gpr116* AEC KO” and “*Gpr116* ECKO”, respectively. In the latter case, pups were induced by tamoxifen gavage of the dam from postnatal day 1 (P1) to P3.

### ROSA^mT/mG^ x Tie2-Cre mice generation

ROSA^mT/mG^ females (Jackson Stock 007576) were crossed to Tie2-Cre males [38] to generate ROSA^mT/mG^ x Tie2-Cre.

### PDGF-B ^ret/ret^ mice generation

PDGF-B ^ret/ret^ mice were generated as previously described [39] and used as a positive control for dextran leakage experiment [40].

### Oxygen induced retinopathy (OIR)

OIR was induced according to the protocol established by Smith *et al*. [33]. Pups at P7 and their nursing dam were transferred to a chamber (A-30274-P, Biospherix) with an oxygen concentration maintained at 75% (ProOx Model 110, Biospherix). At P12, pups and their dam were returned to room oxygen levels and pups were sacrificed at P17.

### Tracer injections

High-molecular weight dextran injection was performed to visualize lumen formation of the retina, as described previously [41], in combination with lectin injection to assess functional vessels and proper blood flow. Briefly, FITC-dextran (2,000 kDa, Sigma Aldrich, FD2000S) and Alexa Fluor 647conjugated lectin (*Griffonia simplicifolia* GS-IB_4_ lectin) were prepared in PBS at the concentration of 25 mg/ml and 1 μg/ml, respectively. P21 *Gpr116* knockout and littermate control were given anesthesia, and intracardiac injection of FITC-dextran and lectin solution was performed. After 3 min, the eyes were enucleated, fixed in 4% paraformaldehyde (PFA), and retinal tissue was processed (see below).

To assess cerebral vascular leakage, 1 kDa Alexa Fluor 555-conjugated cadaverine or 70 kDa tetramethylrhodamine-conjugated dextran (Life Technologies) was injected intravenously into the tail vein in adult mice (1.5 or 18 months). After 2 hours the anesthetized animals were perfused for 5 min with Hanks’ balanced salt solution (HBSS), brains and lung tissues were removed and homogenized in 1% Triton X-100 in PBS (pH 7.2). Brain and lung lysates were centrifuged at 13,000 rpm for 20 min at 4°C and the relative fluorescence of the supernatant was measured on a fluorometer Synergy HT 271167 plate reader (excitation /emission 540/590 nm). After HBSS perfusion, tracer extravasation into brain parenchyma was visualized with a Leica stereomicroscope [40].

### Organ homogenates and FACS

Organs were digested in collagenase (0.5 mg/ml) for 20 min at 37°C. Lungs were digested according to Rawling´s protocol [42] with slight modification. Mice were anesthetized, and perfused with HBSS through the right ventricle of the heart. The trachea was exposed and a 20-gauge blunt needle was inserted into the trachea. A digestion solution (collagenase/dispase, Roche, 0.8 U/ml, DNAse I, Life Technologies, 35 U/ml, and heparin, Sigma, 0.5 mg/ml in PBS) was used to inflate the lungs, which were then minced and exposed to external digestion in the same solution. After inhibition of the collagenase activity by 20% FCS-containing DMEM (Life Technologies), the homogenates were filtered on a 100 micron mesh, pelleted, and resuspended in DMEM containing 0.2% FBS.

To assess the cellular morphology in the lung homogenates, cells were then seeded on glass coverslip, cultivated 24 hours in RPMI medium containing 10% FBS (Life technologies), and fixed in 4% PFA for 5 min at room temperature (RT), permeabilized using permeabilisation buffer (0.1% BSA and 0.05% Triton X-100 in PBS) for 10 min at RT, and incubated with primary antibodies (CD45, BD Pharmingen, 1 hour, RT, 1:500) followed by secondary antibodies (Jackson, 2 hours, RT, 1:500) or phalloidin-Alexa 647 (PromoFluor, Mediqip, 1 hour, RT, 1:1000) as well as Hoechst (Sigma, 1:1000) and mounted in ProLong mounting medium (Life Technologies).

To analyze autofluorescence in homogenates from 4-weeks-old mice, cell suspensions were incubated with Alexa Fluor 780- and BV421-conjugated antibodies against CD45 and CD11b, respectively (eBiosciences, 15 min, RT, 1:100), or Alexa Fluor 780 or BV421 isotope control, respectively (BD Bioscience). Cells were then washed in 10 ml of DMEM with 0.1% FBS, centrifuged, resuspended and sorted using a BD FACSAria III (BD Biosciences). Selection for autofluorescent cells was based on their emission in the green (excitation 488 nm) and red (excitation 561 nm) spectrum. The gated autofluorescent cells were then analyzed for the expression of CD45. Positive cells were gated based upon a threshold set by Alexa 780 isotope control staining. Finally, CD45 positive cells selected previously were investigated for the expression of CD11b. Selection of positive cells was obtained by investigating maximum emission strength of cells stained with a BV421 isotype control.

To analyze autofluorescence analysis in lung homogenates from aged mice, cells were fixed with 1% PFA and analyzed using a BD FACSCantoII (BD Biosciences).

### Brain endothelial cell isolation, culture and staining

Brain endothelial cells (EC) were isolated as described in [22] and [43] with modification. Brain tissue was digested in collagenase (Sigma, 0.5 mg/ml), for 20 min at 37°C, serum-inactivated by adding FBS-containing medium (Life Technologies) and filtered through a 70 μm mesh and centrifuged for 10 min at 1,500 rpm. The pellet was resuspended in DMEM without serum and incubated with CD31 antibody (BD) coupled to Dynabeads (Life Technologies) for 30 min at 37°C. Vascular fragments were pulled down on a DynaMag-2 magnet (Life Technologies), rinsed in DMEM then digested in TrypLE 10X (Life Technologies), for 10 min at 37°C. The vascular fragments were reseeded on coverslips coated with gelatin (Merck) in a 24 well-plate (Falcon), and cultivated in Endothelial Cell Growth Medium2 (Mediqip). Upon reaching confluency, EC were fixed with 4% PFA for 5 min at RT, permeabilized using permeabilisation buffer (0.1% BSA and 0.05% Triton X-100 in PBS) for 10 min at RT, and incubated overnight with primary antibodies (Erg, 1:1000), followed by secondary antibodies (Jackson, 2 hours, RT, 1:500) as well as Hoechst (Sigma, 1:1000) and phalloidin-Alexa Fluor 555 (PromoFluor, Mediqip, 1:1000) and mounted in ProLong mounting medium (Life Technologies).

### Extraction of Bronchoalveolar Lavage Fluid (BALF)

Deeply anesthetized animals were perfused for 5 min with HBSS, before cannulating the trachea with a 0.58 mm diameter polyethylene tube (Cat# 427410, Becton Dickinson) connected to a syringe. 200 μl aliquots of PBS were used to fill the lungs and collected. The samples were cleared by centrifugation for 10 min at 1000 g, before further analysis.

### Measurement of Protein Contents and Saturated Phosphatidylcholine (SatPC)

BALF was obtained as described above. The protein concentration was determined using a BCA Protein Assay (Pierce). For SatPC measurement, lipids were extracted by 10 min incubation of BALF with methanol/chloroform (1:2). After centrifugation for 10 min at 1000 g, the organic phase was collected and evaporated to dryness under a N_2_ flow, prior to analysis with PC Assay kit (Cell BioLabs).

### Western blot analysis of BALF and lungs

For western blots, one snap-frozen lung lobe was lysed in lysis buffer (50 mM Tris-HCl pH 7.5, 150 mM NaCl, 0.5% deoxycholate, 0.5% SDS, 0.1% NP-40, 0.1% Triton X-100, supplemented with PhosSTOP phosphatase inhibitor and Complete protease inhibitor cocktail (both Roche Diagnostics), using a Precellys24 homogenizer and CK14 tubes (Bertin Technologies SAS, Montigny le Bretonneux, France). Tissue debris were removed from the lysates by centrifugation at 4°C for 30 min at 10,000 rpm 10 μg of total proteins were separated by SDS-PAGE on 4–12% gradient gels (Biorad) and transferred to PVDF membranes (Immobilon-P, Millipore). For the detection of proteins, the following primary antibodies were used: goat anti-SP-C, rabbit anti-SP-A (Santa Cruz Biotechnologies, 1:500), rabbit anti-Actin (Cell Signaling Technologies, 1:2000), rabbit anti-calnexin ([44], 1:5000).

### Quantitative RT-PCR analysis on organs and isolated endothelial cells

Total RNA was isolated from frozen whole organs using the RNeasy minikit, and from sorted EC using the RNeasy microkit (QIAGEN). For the P2 lung, first strand cDNA was synthesized from 0.5–1 μg total RNA using iScript cDNA Synthesis Kit (Bio-Rad). Real-Time quantitative PCR (qRT-PCR) was performed using KAPA SYBR FAST qPCR Kit Master Mix (2x) Universal (KAPA Biosystems) in Rotor-Gene Q (Qiagen) Real-Time PCR thermal cycler according to the manufacturers’ instructions. Housekeeping genes were chosen using the TATAA biocenter reference gene panel and GenEx data analysis tool. Expression levels were normalized to the expression of RPL19 and B2m. The primers used were as follows: *Gpr116* (AAGAACAGGACATCCGCTCA/AAAACCTTTCCACGGAGTGC), *SP-A* (TGATCACATGCTGCCTACCA/ATATGGGCACAGGTCTGGAG), *SP-B* (GCAGAACTCTGATCAAGCGG/TGGCATCCTCAGTGGAACAT), *SP-C* (AGCATCCCTAGTCTTGAGGC/CGGACTCGGAACCAGTATCA), *SP-D* (AACACCTGCACCCTAGTCAT/CCTGGAGGTCCACTTAGTCC), housekeeping genes *RPL19* (GGTGACCTGGATGAGAAGGA/TTCAGCTTGTGGATGTGCTC) and *B2m (*CTGACCGGCCTGTATGCTAT/CCGTTCTTCAGCATTTGGAT).

For P4 whole organs and sorted EC, cDNA was synthesized using the iScript cDNA Synthesis Kit (Bio-Rad) and Real-Time quantitative PCR was carried out with the TaqMan® Gene Expression Master Mix (Life technologies) on a CFX96 Touch™ Real-Time PCR Detection System (Bio-Rad). The following probes were used: Gpr116 (Assay: Mm01269028_m1, FAM, spanning exon 17–18 and Assay: Mm01269033, FAM, _m1 spanning exon boundary 2–3, Life Technologies) and Gapdh (Asssay: qMmuCEP0039581, Cy5, Bio-Rad).

### Organ histology

*Gpr116* wild type and knockout mice were anesthetized, and intracardiac perfusion of HBSS was performed. For lung histology, the trachea was exposed and a 20-gauge blunt needle was inserted, perfused with a 4% PFA solution to inflate the lungs, which were post-fixed overnight in 4% PFA solution.

For cryosections, fixed organs were then bathed overnight in a 30% sucrose solution, embedded in OCT (Neg-50, Richard Allan, Thermo Scientific), and sectioned at 10 microns (lung) or 16 microns (brain, liver, kidney) on a CryoSTAR NX70 cryostat (Thermo). For immunofluorescence, sections were thawed, permeabilized for one hour in blocking buffer (1% BSA and 0.5% Triton X-100 in PBS), incubated overnight with primary antibodies, ADRP (Fitzgerald, 1:1000) and CD31 (R&D Systems, 1:1000), washed in PBS and incubated two hours with Alexa Fluor 647-conjugated secondary antibodies (Jackson, 1:1000), or Cy3-conjugated α-smooth muscle actin (Sigma, 1:1000) and Hoechst (Sigma, 1:1000), then mounted in ProLong mounting medium.

For paraffin sections, lungs were included in paraffin and sectioned at 8 microns using a HM355S microtome (Thermo). After deparaffinization, endogenous peroxydases were quenched using 1% H_2_O_2_ and permeabilized, then incubated with primary antibody against CD68 (AbSerotech, 1:1000) for three hours, followed by incubation with HRP-coupled anti-rat secondary antibody (GE Healthcare, 1:1000). Sections were washed, and the signal was revealed with a DAB kit (Vector Laboratories), and counterstained with Mayer´s hematoxylin. Finally, slides were dehydrated and mounted in Neo-Mount (Merck).

For brain vibratome sections, fixed brains were sectioned at 50 microns on a Microm HM650V vibratome (Thermo Scientific). Sections were blocked and permeabilized overnight, incubated 2 days with primary antibodies: CD31, GFAP (Dako, 1:100), Glut-1 (Millipore, 1:300), PDGFRβ (Bioscience, 1:200), ZO-1 (Zymed, 1:200), Alexa Fluor 488-conjugated Claudin-5 (Life technologies, 1:500), VE-cadherin (eBiosciences, 1:200), one day with fluorophore-conjugated secondary antibodies or Cy3-conjugated α-smooth muscle actin (Sigma, 1:1000) and mounted in ProLong mounting medium (Life Technologies, 1:500).

For transmission electron microscopy, lungs were fixed in 2% glutaraldehyde and 1% paraformaldehyde in 0.1 M sodium cacodylate buffer containing 0.1 M sucrose and 3 mM CaCl_2_ (pH 7.4) for 30 minutes at RT, followed by 24 hours at 4°C. The lungs were then rinsed in 0.15 M sodium cacodylate buffer containing 3mM CaCl_2_ (pH 7.4), post-fixed in 2% osmium tetroxide in 0.07 M sodium cacodylate buffer containing 1.5 mM CaCl_2_ (pH 7.4) for 2 hours at 4°C, dehydrated in ethanol followed by acetone and embedded in LX-112 (Ladd, Burlington, VT). Semithin sections were made and stained with toluidine blue and used for light microscopic analysis. Ultrathin section were prepared on a Leica Ultracut UCT (Leica, Wien, Austria) and contrasted with uranyl acetate, followed by lead citrate and examined in a Tecnai 10 transmission electron microscope at 80 kV (FEI Company). Digital images were made using a MegaView III digital camera (Soft Imaging System, GmbH, Münster, Germany).

### Ear whole-mount staining

The outer ear was split in half to remove the auricular cartilage, and the dorsal half was post-fixed overnight in a 4% PFA solution. For immunofluorescence, sections were permeabilized for one hour in blocking buffer (1% BSA and 0.5% Triton X-100 in PBS), incubated overnight with primary antibody (CD31, 1:500), washed in PBS and incubated two hours with Alexa-conjugated secondary antibodies (Jackson, 1:1000), Cy3-conjugated α-smooth muscle actin (Sigma, 1:1000) and Hoechst (Sigma, 1:1000), then flat mounted in ProLong mounting medium.

### Retinal whole-mount staining

Retinas were fixed in 4% PFA in PBS, dissected, permeabilized at 4°C overnight, rinsed in PBS, washed twice in PBlec (1% Triton-X100, 0.1 mM CaCl_2_, 0.1 mM MgCl_2_, 0.1 mM MnCl_2_ in PBS, pH 6.8), and incubated overnight at 4°C with either FITC-conjugated (Sigma-Aldrich #L2895) or Alexa Fluor® 647-conjugated isolectin B4 (Invitrogen), as well as primary antibodies (CD31, NG2, Erg (Abcam), ASMA (Sigma), all 1:1000). After 3 washes in PBS, retinas were incubated for 2 hours at RT with secondary antibodies, 1:400 diluted in PBS, 0.5% BSA and 0.25% Triton X-100. Retinas were flat-mounted in ProLong Gold mounting medium (Life Technologies) and analyzed using a Leica SP8 confocal microscope. Images were assembled using Adobe Photoshop CS5 (Adobe Systems) and the ones shown are either single Z-section or maximum intensity projections of several Z-stacks. Avascular and total retina areas were quantified with ImageJ software (http://rsb.info.nih.gov/ij/).

### *In situ* RNA hybridization

*In situ* RNA hybridization was performed using RNAscope technology (Advanced Cell Diagnostics) following the manufacturer’s protocol with minor modifications. The probe was designed to recognize nucleotide 1892 to 2795, spanning exon 13 to 17, a sequence entirely suppressed in the knockout allele, where from exon 4 to 21 are excised. Briefly, fresh-frozen brains tissues were cut into 16 μm sagittal sections and mounted on SuperFrost Plus glass slides. After dehydration, slides were subjected to RNAscope Multiplex Fluorescent Assay. First, slides were incubated in Pretreat 3 solution for 20 min at RT. RNAscope probes (*Gpr116* and *PECAM)* were then hybridized for 2 h at 40°C, followed by amplification steps according to the manufacturer´s instructions. The fluorescent signal from RNA probes was visualized and captured using a Leica TCS SP8 confocal microscope (Leica Microsystems). All *in situ* hybridization images presented are 2D maximum intensity projections of ~3 μm Z-stacks.

### Confocal microscopy

All confocal images were acquired on a Leica SP8 confocal equipped with a tunable WWL, and processed using Photoshop (Adobe). For quantifications, images were analyzed using ImageJ software (NIH).

### *Gpr116* mRNA expression in published dataset

To identify *Gpr116* mRNA expression levels in the organ-specific isolated EC, we utilized the microarray data set from the published study [45]. The array raw data were downloaded from NCBI GEO database (accession number: GSE47067), and were normalized using the PLIER algorithms (Affymetrix Technical Note. Guide to Probe Logarithmic Intensity Error Estimation. http://affymetrix.com/support/technical/technotesmain.affx). For *Gpr116* mRNA expression in the brain-specific isolated EC, we extracted from the processed data in the supplementary table in the published brain transcriptome [46].

### Statistical analysis

Data are expressed as mean ± SD. In datasets containing two distinct groups, statistical comparisons were performed with the Student’s t-test, and P<0.05 was considered statistically significant. In dataset containing three distinct groups, statistical comparisons among groups were performed using one-way ANOVA followed by Tukey´s post-hoc test and P<0.05 was considered statistically significant. On the figures, the error bars represent SD, and P<0.05 is represented as *, P<0.005 as **, P<0.0005 as ***, P<0.00005 as **** and “ns” stands for “no significant difference”.

## Results

### Analysis of *Gpr116* expression patterns and generation of *Gpr116* knockout mice

We previously identified *Gpr116* as part of the endothelial core transcriptome *in vivo* in mice [22]. This approach relied on the separation of a vascular cluster from the lung into endothelial and non-endothelial (*i*.*e*. epithelial) transcripts, using vascular transcriptomes from other tissues for comparison [22]. Using this approach, potentially erroneous conclusions could be obtained for genes expressed in epithelial cells in the lung and endothelial cells in other tissues [19][21]. While this appears to be the case for Gpr116, its endothelial expression has remained uncertain. However, the recent establishment of organ-specific endothelial transcriptomes [45] confirms *Gpr116* expression in all vascular beds of adult solid organs (Fig 1A). To further support the endothelial expression of *Gpr116*, ROSA^mT/mG^ and Tie2-Cre mice were crossed to obtain ROSA^mT/mG^ x Tie2-Cre offspring. As expected, all cells express a membrane-localized red fluorescence protein (TdTomato) except for the endothelium, where instead the Tie2-Cre promoter leads to expression of a membrane-localized green fluorescence protein (GFP). GFP^+^ cells from 3-weeks-old ROSA^mT/mG^ x Tie2-Cre mice were FACS sorted for each organ, and quantification of *Gpr116* mRNA was performed (Fig 1B). In the case of the brain, transcription profiling of endothelial cells isolated by FACS from Tie2-GFP reporter mice also showed *Gpr116* expression in endothelial cells [46](Fig 1C). Interestingly, the levels of *Gpr116* varied among the different vascular beds, with a higher expression in the brain/heart/lung cluster, as well as in the kidney (defined in ref [45]). To localize *Gpr116* vascular expression in the brain, *Gpr116* mRNA-expressing cells were assessed using the RNAscope *in situ* hybridization technique. For this analysis, we used tissues from Gpr116 knockout mice which are missing the Gpr116 sequence from exon 4 to 21. Since the probe targets exon 13 to 17, these tissues acted as a control for its specificity. We confirmed a general vascular endothelial pattern of expression for *Gpr116* (shown in the cortical sub-region in Fig 1I). Overall, *Gpr116* exhibited a specific, but non-exclusive endothelial distribution.

To delineate the functions of Gpr116 *in vivo*, a new mouse model of genetic inactivation of *Gpr116* was generated. ES cells with 129S6SvEv/C57BL6F1 background were generated through a VelociGene approach, in which a 32 kbp region spanning exons 4 to 21 was replaced by a lacZ lox-Ub1 promoter- EM7 Neomycin-lox cassette (Fig 1D). The recombination efficiency was assessed in the ES cells through a “loss-of-native-allele” assay [35], and a clone with a score of 0.87 was used to generate the *Gpr116* knockout mouse line. Heterozygous and homozygous knockout mice identified by PCR (schematized in Fig 1D, typical results in Fig 1E), were born in strict Mendelian ratios. The inserted transmembrane domain-lacZ (TM-lacZ, [47]) gene was detected in knockout biopsies at the genomic level (Fig 1F). Unfortunately, no LacZ mRNA was detected by qRT-PCR nor by a functional enzymatic activity of the ß-galactosidase, which prevented the intended use of this mouse strain as a reporter of Gpr116 expression (data not shown). The measurement of mRNA from total organs of wild type, heterozygous and knockout postnatal day (P4) littermates confirmed the efficiency of the inactivation strategy. *Gpr116* mRNA was undetectable in knockout animals using primers spanning exon 17 (Fig 1G), whereas heterozygotes presented approximately half of the mRNA levels of the wild type, as expected. Importantly, levels of Gpr116 mRNA coding for exons 2 and 3 were severely reduced in knockout animals, which might explain the absence of expression of the LacZ reporter (Fig 1H).

### *Gpr116* deletion results in a massive pulmonary surfactant accumulation

**Fig 2:**
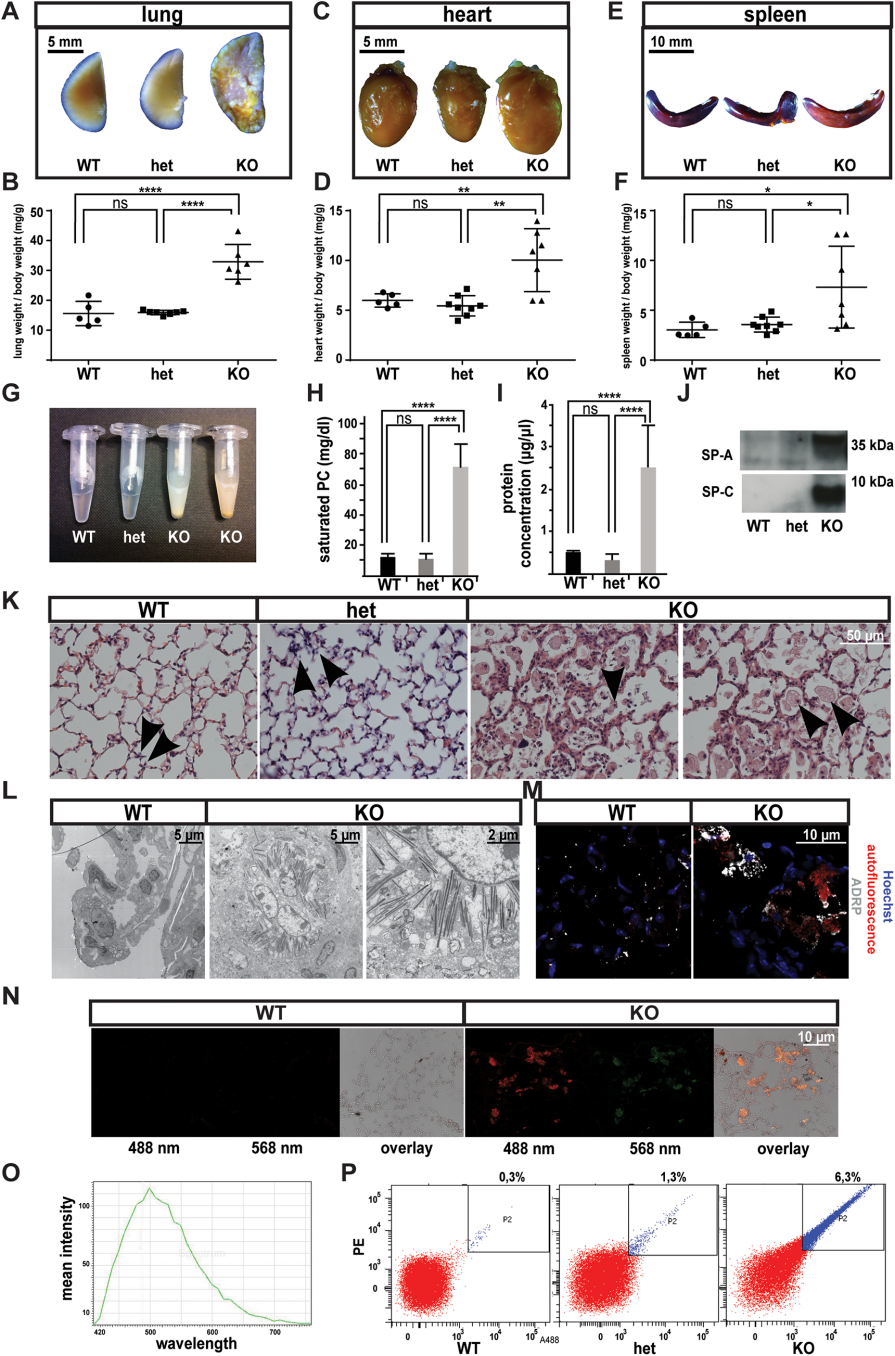
Massive accumulation phenotype in lungs of aged *Gpr116* ^-/-^ mice. A. Bright field image of the inflated lung from *Gpr116* WT, heterozygous and knockout littermates. B. Weights of whole lungs over total body weight from *Gpr116* WT, heterozygous and knockout littermates (n≥5 mice per genotype). C. Bright field images of heart from *Gpr116* WT, heterozygous and knockout littermates. D. Weights of the heart (left) over total body weight from *Gpr116* WT, heterozygous and knockout littermates (n≥5 mice per genotype). E. Bright field images of the spleen from *Gpr116* WT, heterozygous and knockout littermates. F. Weights of the spleen (left) over total body weight from *Gpr116* WT, heterozygous and knockout littermates (n≥5 mice per genotype). G. BALF collected from *Gpr116* WT, heterozygous and knockout littermates (The picture shown is representative of 3 mice for each genotype). H. Quantification of saturated phosphatydilcholine in BALF by ELISA (n = 3 mice per genotype). I. Quantification of protein content in BALF by BCA assay (n = 3 mice per genotype). J. Surfactant proteins detection in BALF by western blot. Molecular weights are indicated on the right. (n = 2 mice per genotype). K. Bright field images of the lung, after hematoxylin and eosin staining. The black arrowheads indicate alveolar macrophages (the image is representative of 4 mice for each genotype). L. Electron microscopy view of *Gpr116* wildtype and knockout lungs (n = 2 mice for each genotype). M. Confocal images of lung sections stained with ADRP (white) and nuclear stain (Hoechst, blue). Note that a red autofluorescent signal appears in knockout lungs. (the image shown is representative of 2 mice for each genotype). N. Confocal images of lung sections stained with nuclear marker Hoechst (blue) to show autofluorescent cells accumulated in the alveolar space, either in the green or red channel (the image is representative of 3 mice for each genotype). O. Autofluorescence emission spectrum of macrophages in the old knockout lung, upon 405 nm excitation (the image is representative of 2 mice). P. Detection of autofluorescent cells from *Gpr116* knockout lung by FACS (n = 2 mice per genotype)

Despite having expected numbers at birth, *Gpr116* knockout mice were affected by a higher rate of spontaneous death than wild type littermates, starting around 14 months of age (data not shown). Autopsies of aged mice (between 12 and 18 months) revealed a striking change in appearance of several organs. The most prominent phenotype was observed in the lungs of the knockout animals, with a strong increase of lung size and weight, as well as pale coloring (Fig 2A and 2B). Heart and spleen were also frequently enlarged in old knockout animals (Fig 2C to 2F). Interestingly, the onset of those phenotypes was noticeable rather early as evidenced in one-month-old animals (S1 Fig). Bronchoalveolar lavage fluid (BALF) of knockout mice was drastically altered in appearance, being whitish and cloudy compared to the rather transparent wild type BALF, and resembling the change in overall appearance of the lungs (Fig 2G). Analysis of the knockout BALF demonstrated an increase in saturated phosphatidylcholine (Fig 2H), total protein content (Fig 2I) and surfactant proteins SP-A, and SP-C (Fig 2J), compared to littermates controls.

The changes in surfactant levels were accompanied by the abundant presence of phagocytic cells in the airspace. Morphological analysis by electron microscopy, as well as hematoxylin-eosin staining, revealed dilated cells that were filled with phagocytic material and were often occupying a major proportion of the airspace (Fig 2K and 2L). ADRP staining indicated that the accumulated material inside the cells was at least partially composed of lipids (Fig 2M), and the cells resembled histologically “foamy” macrophages that are typically present. in atherosclerotic lesions. Furthermore, the foamy macrophages exhibited a strong autofluorescence, in the visible range, which was quenched by glycerin treatment (visible range is 435 nm—724 nm: spectrum shown in Fig 2N and the appearance of the cells shown in Fig 2O). Observing the macrophages accumulating in aortic roots of *ApoE* null mice, identical autofluorescent properties were demonstrated by these cells (data not shown). Therefore, we hypothesized that such autofluorescent properties could be used as a quantitative tool. Analysis of unstained cells from *Gpr116* knockout lung homogenates sorted by FACS, indeed displayed an increase in large, autofluorescent cells (Fig 2P).

The severity of the pulmonary phenotype in aged animals prompted us to examine early lung dysfunction. To preclude the possibility of an early surfactant deficiency that could lead to a complex phenotype later in life, we assessed surfactant mRNA level in P2 mouse lung and found no changes in *Gpr116* knockouts compared to wild type littermates (S2A Fig). To distinguish among the various features of the pulmonary phenotypes in *Gpr116* knockouts, we assessed their emergence in postnatal lungs. At 2 weeks, knockout lungs appeared normal, the alveolar morphology was normal and foamy macrophages could not be detected. However, the BALF from 2-weeks-old knockout mice was already whitish in color and showed a clear increase in protein content (S2B Fig). This alteration of BALF persisted at 3 and 4 weeks of age and, at this point, was accompanied by changes in the size of alveolar macrophages which gradually increased with age (S2C and S2D Fig). At 4 weeks, cells in the alveolar space of the knockout lungs started to display the typical autofluorescent pattern described in the older lungs, and the majority of these cells were CD45-positive immune cells (S2E Fig). The autofluorescent, CD45-positive cells were prominent in single cell suspensions of 4 weeks-old-mice lungs (S2F Fig). Accordingly, when those suspensions were FACS sorted, Gpr116 knockout mice displayed a larger population of autofluorescent cells compared to Gpr116 heterozygotes or Gpr116 wild type mice (S2G Fig). The gated autofluorescent cells selected from the knockout population were analyzed for the expression of CD45 and the results indicate that immune cells contribute to the majority of the autofluorescence (92.4% ±1.9) (S2H Fig). Furthermore, approximately half of the cells were identified as CD11b-positive, a pan-macrophage marker (S2I Fig).

The involvement of macrophages as well as the various cell types expressing Gpr116 prompted us to employ a genetic tool, consisting of a Gpr116 allele where exon 17 has been flanked by LoxP sequences [19] to delete Gpr116 gene in a cell-specific manner. Inactivation of the floxed allele by Cre recombinase expressed from the type II pneumocyte-specific SP-C promoter, termed “Gpr116 AEC KO” mice, resulted in altered surfactant at 4 weeks, with an increase in protein content (S2J Fig). The alveolar macrophages in Gpr116 AEC KO lungs appeared enlarged in size and contained a cytoplasm full of phagocytosed elements. However, these features seemed delayed when compared to the full knockout animals, both at the level of the protein content in BALF (2.11 μg/μL ± 0.2, for the full knockout, vs 1.04 μg/μL ± 0.1, P<0.005), and the average size of macrophages (20.2 μm^2^ ± 0.6, n = 399 for the full knockout, vs 9.3 μm^2^ ± 0.3, n = 248 for the SP-C Cre knockout, P< 0.0001). Overall, the SP-C Cre-mediated Gpr116 knockout phenocopied the main features of the full knockout, in regards to the lung phenotype, despite a later progression that may be a result of incomplete gene inactivation. Nonetheless, these results demonstrate that the lack of Gpr116 in the type II pneumocytes caused the surfactant defect. On the contrary, genetic ablation of Gpr116 in the endothelium (“Gpr116 ECKO”) did not result in any obvious alteration in alveolar morphology (S2K Fig). Taken together, these results indicate that the loss of Gpr116 leads to an early and rapid surfactant deregulation, which progresses into a massive macrophage infiltration and severe pulmonary emphysema in aged knockout mice.

### *Gpr116* deletion does not disrupt vascular patterning and perfusion

**Fig 3:**
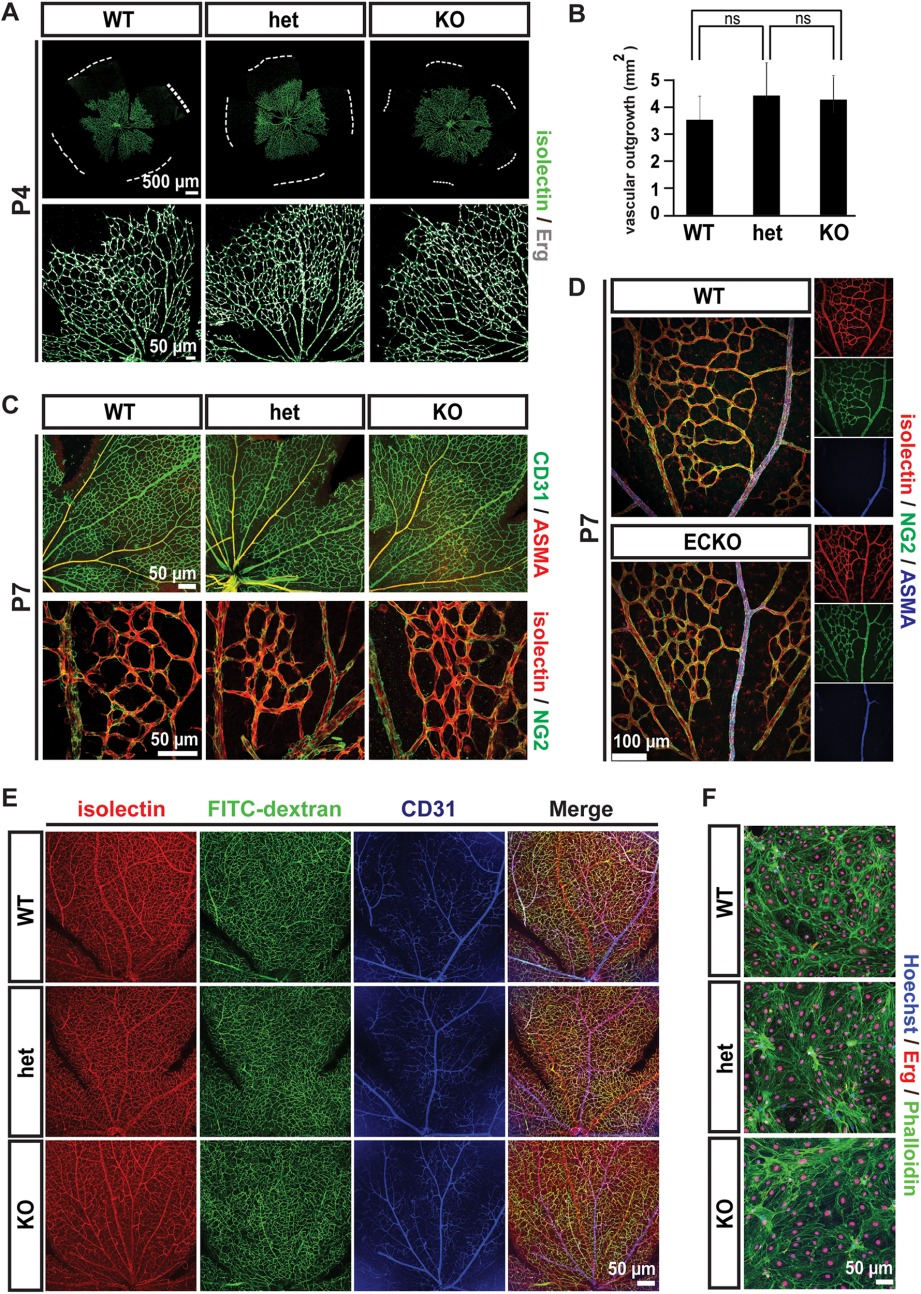
Retinal vascular patterning in *Gpr116* ^-/-^ mice. A. Vascular network in P4 retinas. Dashed line indicates the limits of the retina (the picture shown is representative of at least 5 mice for each genotype). B. Quantification of the retinal vascular outgrowth at P4 (n = 5 for WT, n = 12 for heterozygotes and n = 6 for knockout). C. Vascular patterning in P7 retinas from *Gpr116* WT, heterozygous and knockout littermates. Isolectin (red), CD31 (green) and Erg (grey) were used to visualize endothelium, and NG2 (green) and ASMA (red) to detect mural cells (the images shown are representative of 3 mice for each genotype). D. Vascular patterning in P7 retinas from *Gpr116* ECKO and littermates controls. Isolectin (red) is used to visualize endothelium, and NG2 (green) and smooth muscle actin α (ASMA, blue) to detect mural cells (the images show are representative of 2 mice per genotype). E. Isolectin (red) and FITC-dextran (green) distribution in P21 retinas from *Gpr116* WT, heterozygous and knockout littermates. CD31 (green) is used to stain the endothelium, and nuclei are stained with Hoechst (blue) (the images shown are representative of 3 mice per genotype). F. Monolayers formed by isolated endothelial cells from *Gpr116* WT, heterozygous and knockout brain. Endothelial cells (CD31) and nuclei (Hoechst) are indicated in green and blue, respectively (the pictures shown are representative of 3 mice for each genotype)

The early and broad expression pattern of *Gpr116* in the endothelium has yet to be linked to a specific vascular role or function. *Gpr116* knockout mice do not display any overt defect in early blood vessel patterns generated by sprouting angiogenesis, as evidenced by examination of the retinal vasculature. The vascular networks at P4 were indistinguishable in morphology (Fig 3A), and occupied equivalent areas in wild type, heterozygous and knockout retinas (Fig 3B). Additionally, the subsequent recruitment of pericytes and arterio-venous differentiation in retinas appeared normal at P7 (Fig 3C). Endothelial-specific ablation of Gpr116 (Gpr116 ECKO) animals showed normal retinal vascular patterning and mural cell recruitment assessed by immunofluorescence staining in NG2 and ASMA (Fig 3D). Additionally, Gpr116 knockout did not exhibit overt abnormalities in the CNS vasculature (S3A and S3B Fig), or other vascular beds (intestinal villi, S3C Fig, kidney glomerulus, S3D Fig, and outer ear, S3E Fig). To determine if the vessels lacking Gpr116 were functional, the distribution of a 2,000 kDa dextran tracer was assessed. After 3 minutes of circulation, FITC-dextran was evenly distributed throughout the entire retinal vascular bed, from arteries to capillaries and veins, and from the central optic nerve to the periphery (Fig 3E). These results indicate a proper perfusion of the retinal vascular network and, given the large size of the tracer, precludes any significant lumen defect in *Gpr116* knockout mice. A proper perfusion and lumen formation were also verified in various organs including brain cortical vessels (S3F Fig), liver (S3G Fig), intestine (S3H Fig), and glomeruli (S3I Fig). Endothelial cells isolated from the brain of knockout animals established a cell monolayer with a density and an actin cytoskeleton indistinguishable from the pattern of the wild type control (Fig 3F). Together, these data suggest that Gpr116 is dispensable for retinal vasculature development and function *in vivo*, and the establishment of brain endothelial cell monolayer *in vitro*.

### *Gpr116* deletion leads to blood-brain-barrier disruption

**Fig 4:**
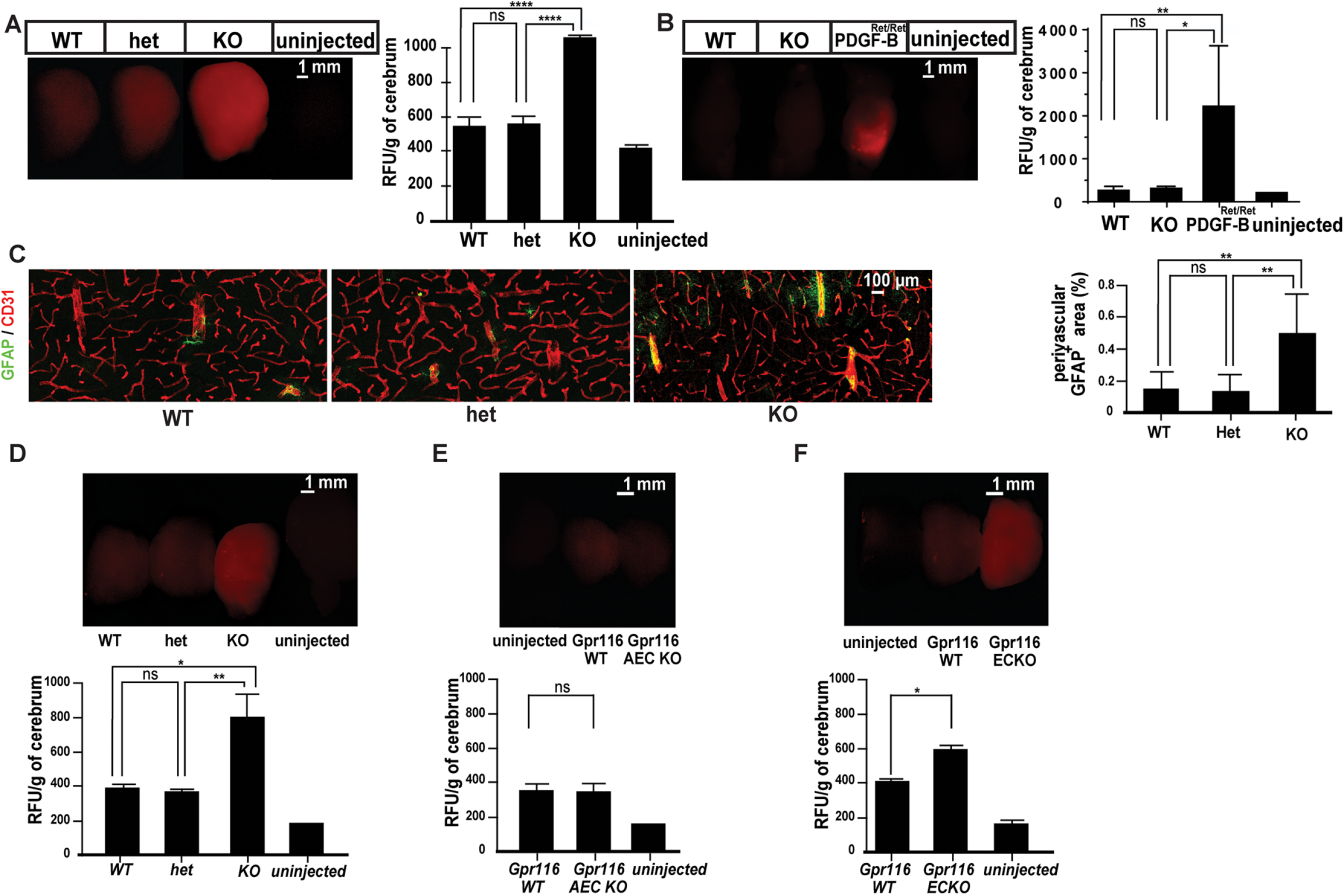
Blood brain barrier breakdown in *Gpr116* ^-/-^ mice. A. Whole brain images taken after 1kDa cadaverine perfusion (left) and associated quantification of extravasated cadaverine (right) in aged *Gpr116* WT, heterozygous and knockout mice (n≥5 mice for each genotype). B. Whole brain images taken 70 kDa tetramethylrhodamine dextran perfusion (left) and quantification of extravasated tracer (right) in *Gpr116* WT and heterozygous and *Gpr116* ECKO mice (n = 3 for wild type and ECKO, n = 2 for *PDGF-B* ^*ret/ret*^, n = 1 for uninjected control). C. Confocal images of cerebral cortex from aged *Gpr116* WT, heterozygous and knockout mice. Astrocytes (GFAP) appear in green, endothelial cells (CD31) in red (the images are representative of 4 mice per genotype) and associated quantification of perivascular associated astrocytes in aged *Gpr116* WT, heterozygous and knockout mice (n = 4 mice for each genotype, 2 sections at least quantified per genotype). D. Whole brain fluorescence images taken after Alexa 555-cadaverine circulation (upper) and quantification of extravasated cadaverine (lower) in 1.5-month-old *Gpr116* knockout (n = 3 mice per genotype). E. Whole brain fluorescent images taken after cadaverine circulation (upper) and associated quantification of extravasated cadaverine (lower) in 2-months-old *Gpr116* AEC KO (n = 6 mice per genotype). F. Whole brain fluorescent images taken after cadaverine circulation (upper) and quantification of extravasated cadaverine (lower) in 2-months-old *Gpr116* ECKO (n = 7 mice per genotype)

To provide a primary assessment of the barrier properties of the Gpr116 deficient vasculature, we injected a 1 kDa fluorescent tracer, Alexa Fluor 555-cadaverine, and examined its tissue distribution. Alexa Fluor 555-cadaverine extravasates into most tissues, but normally is excluded from the CNS, the vasculature of which is endowed with specific barrier properties, termed the blood-brain barrier (BBB). As shown in Fig 4A, Gpr116 deficient vessels in 12-months-old animals failed to fully retain the 1 kDa Alexa Fluor 555-cadaverine tracer after tail vein injection, leading to tracer accumulation in the brain parenchyma. However, the BBB impairment was restricted to small molecular weight tracers. Lysine-fixable, 70 kDa tetramethylrhodamine-conjugated dextran did not accumulate into the knockout brains, in comparison to PDGF-B ^Ret/Ret^ mice where the loss of pericytes leads to leakage of high molecular weight molecules (Fig 4B)[40]. Consistent with sustained BBB impairment, immunostaining for the intermediate filament protein GFAP in order to visualize astrocytes, showed increased GFAP-positive areas around blood vessels in 18-months-old knockouts, suggesting astrogliosis (Fig 4C). Tracer leakage across the BBB could also be detected in young (1.5 month) full knockout animals (Fig 4D). Interestingly, no BBB leakage was observed in 2-months-old *Gpr116* AEC KO mice (Fig 4E) which showed a strong lung phenotype (S2K Fig). These data argue against the BBB leakage in *Gpr116* knockout mice as a secondary consequence of the pulmonary pathology, and raise the possibility that endothelial cell-driven Gpr116 is responsible for the BBB leakage. Genetic ablation of Gpr116 under the control of VE-Cadherin (VE-cad:Cre ER [37]**)**, an endothelial specific inducible promoter, resulted in a significant increase of the 1 kDa Alexa Fluor 555-cadaverine leakage in the 2-months-old mouse brains (Fig 4F). Of note, pericyte coverage (S3B Fig) and patterning of the endothelial junctions (S4 Fig) were comparable to littermate control in the knockout brain cortex. These results establish the first evidence for endothelial cell-autonomous function(s) of Gpr116 *in vivo*.

### *Gpr116* deletion modulates pathological angiogenesis in retina

Pathological angiogenesis, especially in ischemic situations, is characterized by formation of dilated and leaky vessels, driven by increased growth factor production. In mice, the oxygen-induced retinopathy (OIR), mimicking human Retinopathy of Prematurity, is a commonly adopted model of induction and study of pathological angiogenesis [33]. Altered responses in the OIR model have been observed in several mutants with normal developmental angiogenesis, suggesting that pathological and developmental angiogenesis may, in part, engage different mechanisms [48][49][50], leading us to ask whether Gpr116 might play a role in pathological neovascularization.

**Fig 5:**
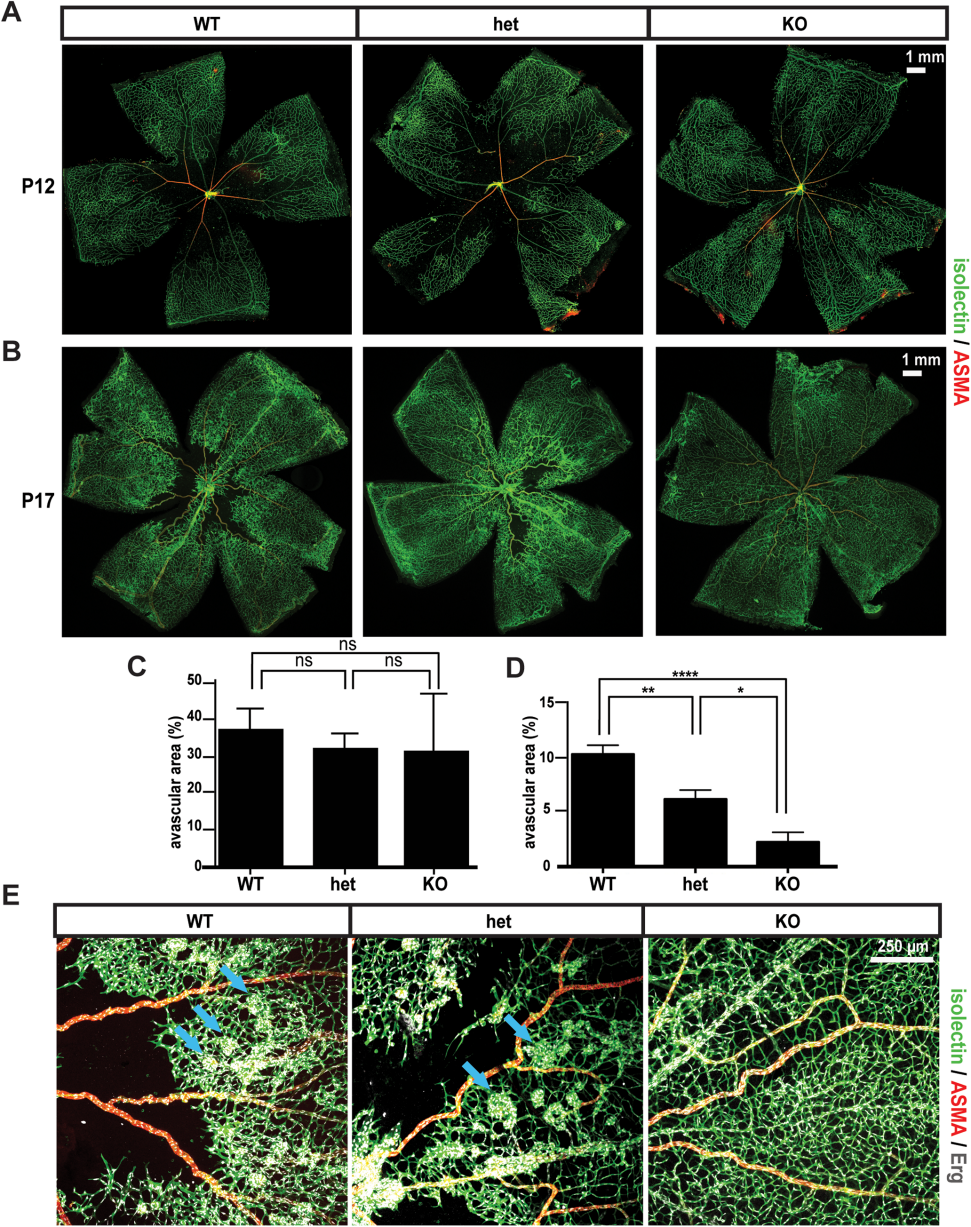
Normalized pathological angiogenesis in *Gpr116* ^-/-^ retinas. A. Confocal images of post-OIR retinas from *Gpr116* WT, heterozygous and knockout littermates at P12 (the images shown are representative of 5 mice per genotype). B. Confocal images of post-OIR retinas from *Gpr116* WT, heterozygous and knockout littermates at P17 (the images shown are representative of 5 mice per genotype). C. Quantification of the avascular area on the post-OIR retinas from *Gpr116* WT, heterozygous and knockout littermates at P12 (n = 5 mice at least per genotype). D. Quantification of the avascular area on the post-OIR retinas from *Gpr116* WT, heterozygous and knockout littermates at P17 (n≥7 mice at least per genotype). E. Confocal images of post-OIR tufts (blue arrows) in *Gpr116* WT, heterozygous and knockout littermates at P17 (the images shown are representative of 5 mice per genotype)

P7 pups were, therefore, submitted to a classical OIR protocol: pups were exposed to 75% hyperoxia from P7 until P12 [33][51], to normal air between P12-17, followed by retinal analysis at P17. As expected, the wild type retinas showed large areas of vaso-obliteration (Fig 5A) and formed pathological neovascular tufts (Fig 5E). In knockout mice, however, despite a stereotypical vaso-obliteration around the optic nerve at P12 [33](Fig 5A, quantified in Fig 5C), physiological revascularization occurred much faster than in wild type retinas, causing an almost complete recovery of the avascularization at P17 (Fig 5B, quantified in Fig 5D). Interestingly, this rapid vascular regrowth was accompanied by an almost complete vascular normalization, with almost undetectable intra-vitreal pathological tufts (Fig 5E). Finally, heterozygous mice, in which vessels express only half of the normal amount of Gpr116, showed an intermediary phenotype with the ratio of the avascular area over the total area reflecting a value midway between knockout and wild type retinas, suggestive of a dose-dependent effect of Gpr116 during pathological angiogenesis.

## Discussion

Most adhesion receptors lack defined ligands and known downstream signaling pathways. This limits the ability to validate the functional consequences of the mutagenesis of the receptors *in vivo* to mRNA and protein expression. This limitation makes it particularly important to confirm phenotypic results by several convergent and independent knockout approaches.

In the case of Gpr116, two different gene deletion strategies have recently been applied in order to generate knockout mice. Two full knockouts have been reported in which exon 2 was targeted. Exon 2 encodes the signal peptide, and its deletion would therefore be expected to inhibit translocation of a nascent protein to the endoplasmic reticulum and further trafficking to the cell membrane [21][20]. In another approach, the mutation deletes exon 17, which encodes one of the transmembrane domains [19]. The knockout model reported here is an extensive, constitutive knockout encompassing exon 4 to part of exon 21 of the *Gpr116* gene, which deletes the potential extracellular adhesion domains, the GPS, as well as all transmembrane domains and a large portion of the C-terminal intracellular domain.

Using this extensive knockout model, we also found that Gpr116 deficiency led to a specific pulmonary phenotype in adult mice, characterized by a profound alteration of surfactant composition, with increased protein and saturated PC content. The other remarkable feature of knockout lungs was a massive accumulation of hypertrophic, and sometimes necrotic, macrophages. These observations complement the previously published findings regarding the *Gpr116* gene deletion in mice aged two months or more, where lipid accumulation is associated with enlarged macrophages and alveoli. Moreover, we describe previously unappreciated functions of Gpr116 in vascular homeostasis and pathophysiology.

The lung dysfunction occurred in an age-dependent fashion in young knockout animals. Previous models identified some transcriptionally altered genes in late embryonic lung (RyR2, [19], and inflammatory cytokines, [52]). The first regulated surfactant components were noted at one (SatPC, [20]) and two weeks (SP-A,[21]) of age. The more extensive evidence showed that, at 4 weeks, the surfactant protein and lipid composition was deregulated. However, the precise sequence of the defects varies among the models, leading to two different interpretations of the pathway regulated by Gpr116. In the exon 17 mutant, satPC synthesis is increased, in line with an overactivation of the *de novo* pathway. In the exon 2 mutants, all surfactant proteins are increased in BALF, followed at 6 weeks by a profound modification in the phospholipid composition of the surfactant, compatible with an alteration of the surfactant recycling pathway. It is important to note that, at 4 weeks, the alveolar structure is already modified [19] and the foamy macrophage phenotype is prominent, indicating that the phenotype is already quite severe (S2C Fig). Our results demonstrate that *Gpr116* knockouts already have a clear surfactant alteration at 2 weeks, which was progressively followed by an accumulation of enlarged macrophages at 3 weeks. Here, we propose a time window in which each component of the lung phenotype can be studied. The initial surfactant deregulation occurs during the first two postnatal weeks, and thus determining whether the *de novo* or the recycling pathway is affected could be explored at those time points. It is noteworthy that the onset of surfactant alteration also clearly preceded the macrophage accumulation and it is consistent with the idea that macrophage enlargement is a secondary event. This concept is reinforced by the fact that Gpr116 deletion in type II pneumocytes, using a specific SP-C Cre promoter, recapitulates the lung abnormalities (show herein at one month, as well as in one year old-mice in ref [21]), albeit with a somewhat diminished severity in the inducible mouse model.

The range of cell types in which Gpr116 plays a critical role still remains to be fully determined. It is all the more difficult to predict in the absence of defined modules downstream of the receptor. In this regard, the importance of Gpr116 in the vascular endothelium, one of the major sites of Gpr116 expression as demonstrated both herein, as well as in previous work, remains enigmatic.

We confirmed previous reports showing the extensive expression of Gpr116 in vascular endothelium, including in the lungs, where its presence in the endothelium in addition to the epithelium has been challenged [19].

Consistent with this extensive vascular expression, the loss of Gpr116 caused a vascular leakage in the brain of 1.5-month-old animals. The absence of any leakage in 2-months-old *Gpr116* AEC KO mice argues against a systemic vascular effect driven by the pulmonary surfactant accumulation. The leakage persisted in older animals, where it correlated with increased astrogliosis. The cause of the leakage, which was restricted to small molecular weight molecules and was of moderate amplitude compared to another leakage model where the BBB opens as a consequence of pericyte deficiency [40], has not been explored in the present study. It is not likely that the BBB disruption in *Gpr116* knockout mice was caused by increased transcytosis, which affects transport of large molecular weight tracers. However, major alterations in the junction distribution of the CNS endothelial cells were not detected and thus, could not completely rule out the former explanation.

The second vascular phenotype that we found in *Gpr116* knockouts was a strikingly “normalized” appearance of the retinal vasculature after OIR challenge. This normalization occurred after a phase of vaso-obliteration comparable to that of wild type controls, indicating that Gpr116 might not impinge on oxygen sensing and the NO synthase pathways. The revascularization of the knockout retina happened much faster than the wild type retina, but did not develop the pathological tufts that are a hallmark of this challenge model and the feature of the equivalent syndrome in premature human infants. The complete revascularization of avascular areas argued against a strong anti-angiogenic activity of Gpr116, as seen in the case of severe blockade of VEGF [53]. The absence of tuft formation is, in turn, one of the more remarkable findings in comparison with previously described OIR phenotypes (such as in [54]), and seems as extensive as the A2A receptor and N-CAM mutants [49][50]. It is also the only phenotype reported to date in *Gpr116* knockouts that presents a graded response, with intermediate effect in the heterozygous littermates. The background of the Gpr116^*Δexon17*^ mice (BALB/c, which is highly resistant to OIR) precluded a confirmation that the recovery is an intrinsic endothelial property. Therefore, the role of an accessory cell type, such as astrocytes, Müller cells or retinal pigmented epithelium cannot be ruled out at this point [55]. Furthermore, even if the results were to be verified using *Gpr116* knockout backcrossed on a C57BL6 background, a potential neighboring effect of modifying genes would be very hard to eliminate. Only the age at which pups are analyzed (P17) argues against the OIR recovery as a secondary consequence of the lung phenotype.

In conclusion, our results strengthen the description of the critical role played by Gpr116 in surfactant regulation: the lack of Gpr116 in type II pneumocytes results in a fast alteration of surfactant composition, with onset occurring at around 1 week after birth. The accumulation of enriched surfactant leads to secondary events where the accumulation of foamy, lipid overloaded macrophages is prominent.

Our work also provides a foundation for exploring the function of Gpr116 outside of the pneumocytes. The severity of the lung phenotype implies that a number of features of full knockout mice might be derived from the general pathological situation in the lung. Still, use of young full knockout, as well as cell type-specific knockout, provides means to untangle the discrete functions of the receptor in different cell types. As a proof-of-concept, we show here that Gpr116 controls a number of functions of the vascular endothelium, one of its main sites of expression. As the vascular functions assessed here are CNS-specific, we cannot yet draw global conclusions about endothelial Gpr116 functions. Clearly, other vascular challenges to *Gpr116* knockout are warranted, as are more specific studies of the mechanisms of vascular permeability and angiogenic sprouting. As for the lung phenotype, ligand identification and distribution as well as discerning the regulated molecular pathways will be critical in order to understand the role of Gpr116 in vascular endothelium.

## Supporting Information

S1 Fig Heart and spleen enlargement in young Gpr116^-/-^ mice. A. Bright field image of the heart from 1-month-old Gpr116 WT, heterozygous and knockout littermates. B. Weights of the heart over total body weight from 1-month-old Gpr116 WT, heterozygous and knockout littermates (n≥4 mice per genotype). C. Bright field image of the spleen from 1-month-old Gpr116 WT, heterozygous and knockout littermates. D. Weights of the spleen over total body weight from 1-month-old Gpr116 WT, heterozygous and knockout littermates (n≥4 mice per genotype).
(TIF)

S2 Fig Sequential development of pulmonary impairment in Gpr116^-/-^ mice. A. Quantification of surfactant mRNA by RT-PCR in lungs from Gpr116 WT, heterozygous and knockout littermates at P2 (n≥4 mice per genotype). B. BALF appearance and protein content quantified by BCA in 2-, 3- and 4- weeks-old animals. Number of mice is indicated in the figure. C. Bright field views of the lung stained with CD68 (HRP) and eosin. The red arrows indicate abnormal macrophages (the pictures shown are representative of 2 mice for each genotype). D. Electron microscopy image of lung from 1-month-old Gpr116 WT and knockout animals. The red arrows indicate abnormal macrophages (the pictures shown are representative of 2 mice per genotype). E. Confocal images of alveoli from 1-month-old Gpr116 WT, heterozygous and knockout mouse lung. On the left panel, the tissue structure is delineated by phalloidin-Alexa 647 (white). On the right panel, immune cells appear in white (CD45). On both panel, cellular autofluorescence is shown in green, and nuclei in blue (Hoechst). A high magnification merged view is presented in the low right corner of each panel (the image is representative of 3 mice for each genotype). F. Confocal images of pre-FACS cells purified from 1-month-old Gpr116 WT, heterozygous and knockout mouse lungs. On the left panel, the cell cytoskeleton is delineated by phalloidin-Alexa 647 (white). On the right panel, immune cells appear in white (CD45). On both panel, cellular autofluorescence is shown in green, and nuclei in blue (Hoechst) (the image shown is representative of 3 mice for each genotype). G. Pseudo-colored scatter plots from FACS purified single cell suspensions from 4-weeks-old-mouse lung. Cell suspensions were incubated with Alexa 780- and BV421-conjugated antibodies against CD45 and CD11b, respectively. Selection for autofluorescent cells is based on their emission in the green (excitation 488 nm) and red (excitation 561 nm) spectrum. Note that Gpr116 knockout mice display a larger population of autofluorescent cells compared to Gpr116 heterozygotes or Gpr116 WT mice (the plot is representative of 3 mice for each genotype). H. The gated autofluorescent cells selected in (G) are analyzed for CD45 expression. CD45- positive cells are gated based upon a threshold set by Alexa 780-isotope control staining. I. CD45+ cells selected in (H) were investigated for CD11b expression. A selection of CD11b-positive cells was obtained by investigating maximum emission strength of cells stained with a BV421 isotype control. Note that the cells display a high autofluorescence in blue (excitation 405 nm). J. BALF appearance and protein content in 1-month-old Gpr116 AEC KO (n = 4 mice per genotype). K. Bright field views of lung from 1-month- or 2-months-old Gpr116 Gpr AEC KO (top rows) and 6-months old Gpr116 ECKO (lower row) stained with hematoxylin and eosin. View from 3-months-old lung from full knockout animal is displayed for comparison (the pictures shown are representative of 2 mice for each genotype).
(TIF)

S3 Fig Development of central and peripheric vascular beds in Gpr116^-/-^ mice. A. Vasculature of the 1-month-old brain cortex from Gpr116 WT, heterozygous and knockout littermates. Glut-1 (green) is used to visualize endothelium, and ASMA (red) to distinguish the major vessels (the images shown are representative of 2 mice per genotype). B. Vasculature of the 1-month-old brain cortex from Gpr116 WT, heterozygous and knockout littermates. CD-31 (green) is used to visualize endothelium, and PDGFRβ (red) to reveal the pericytes coverage (the images shown representative of 2 mice per genotype). C. Vasculature of the 1-month-old intestine villi from Gpr116 WT, heterozygous and knockout littermates. CD-31 (green) is used to visualize endothelium, and ASMA (red) to distinguish the major vessels (the images shown are representative of 2 mice per genotype). D. Vascular beds in 1-month-old kidney glomerulus from Gpr116 WT, heterozygous and knockout littermates. CD-31 (green) is used to visualize endothelium, and ASMA (red) to distinguish the afferent arteriole (the images shown are representative of 2 mice per genotype). E. Vascular beds in 1-month-old outer ear from Gpr116 WT, heterozygous and knockout littermates. CD-31 (green) is used to visualize endothelium, and ASMA (red) to distinguish the major vessels (the images shown are representative of 2 mice per genotype). F. Isolectin (red) and FITC-dextran (green) distribution in P21 cerebral cortex from Gpr116 WT, heterozygous and knockout littermates. CD31 (grey) is used to visualize the endothelium (the images shown are representative of 3 mice per genotype). G. Isolectin (red) and FITC-dextran (green) distribution in P21 liver from Gpr116 WT, heterozygous and knockout littermates. CD31 (grey) is used to visualize the endothelium (the images shown are representative of 3 mice per genotype). H. Isolectin (red) and FITC-dextran (green) distribution in P21 intestinal villi from Gpr116 WT, heterozygous and knockout littermates (the images shown are representative of 3 mice per genotype). I. Isolectin (red) and FITC-dextran (green) distribution in P21 kidney glomerulus and afferent arteriole from Gpr116 WT, heterozygous and knockout littermates (the images shown are representative of 3 mice per genotype).
(TIF)

S4 Fig Vascular junction pattern in Gpr116^-/-^ mouse brain cortical vessels. A. Adherens junction staining in 1-month-old brain cortex from Gpr116 WT, heterozygous and knockout littermates. VE-cadherin (red) is used to visualize adherens junctions, and podocalyxin (green) to delineate the vessels. A higher magnification of VE-cadherin is shown on the right side of the figure (the images shown are representative of 2 mice per genotype). B. Tight junction patterning in 1-month-old brain cortex from Gpr116 WT, heterozygous and knockout littermates. Claudin-5 (red) is used to visualize tight junctions, and CD31 (green) to delineate the vessels. A higher magnification of the claudin-5 is shown on the right side of the figure (the images shown are representative of 2 mice per genotype). C. Tight junction staining in 1-month-old brain cortex from Gpr116 WT, heterozygous and knockout littermates. Another tight junction marker, Zo-1 (red) is used and CD31 (green) to delineate the vessels. A higher magnification of the ZO-1 is shown on the right side of the figure (the images shown are representative of 2 mice per genotype).
(PDF)

S5 Fig Graphical abstract. (PDF)

## Abbreviations

ADRP: Adipophilin
AEC: alveolar epithelial cell
ApoE: Apolipoprotein E
ASMA: α Smooth Muscle Actin
BALF: Bronchoalveolar Lavage Fluid
BBB: Blood Brain Barrier
BCA: bicinchoninic acid assay
DMEM: Dulbecco´s Modified Eagle´s Medium
EC: endothelial cells
GFAP: glial fibrillary acidic protein
HBSS: Hank´s Balanced Salt Solution
het: heterozygote
KO: knockout
mT/mG: membrane-Tomato/membrane-Green
OIR: oxygen-induced retinopathy
PFA: paraformaldehyde
Sat PC: saturated phosphatidylcholine
TM-lacZ: transmembrane domain-lacZ
VE-Cad: VE-Cadherin
