# Supplementary figures and images for: Gpr116 Receptor Regulates Distinctive Functions in Pneumocytes and Vascular Endothelium
Source: PLoS One. 2015 Sep 22;10(9):e0137949. doi: 10.1371/journal.pone.0137949 (PMC4579087; doi:10.1371/journal.pone.0137949)

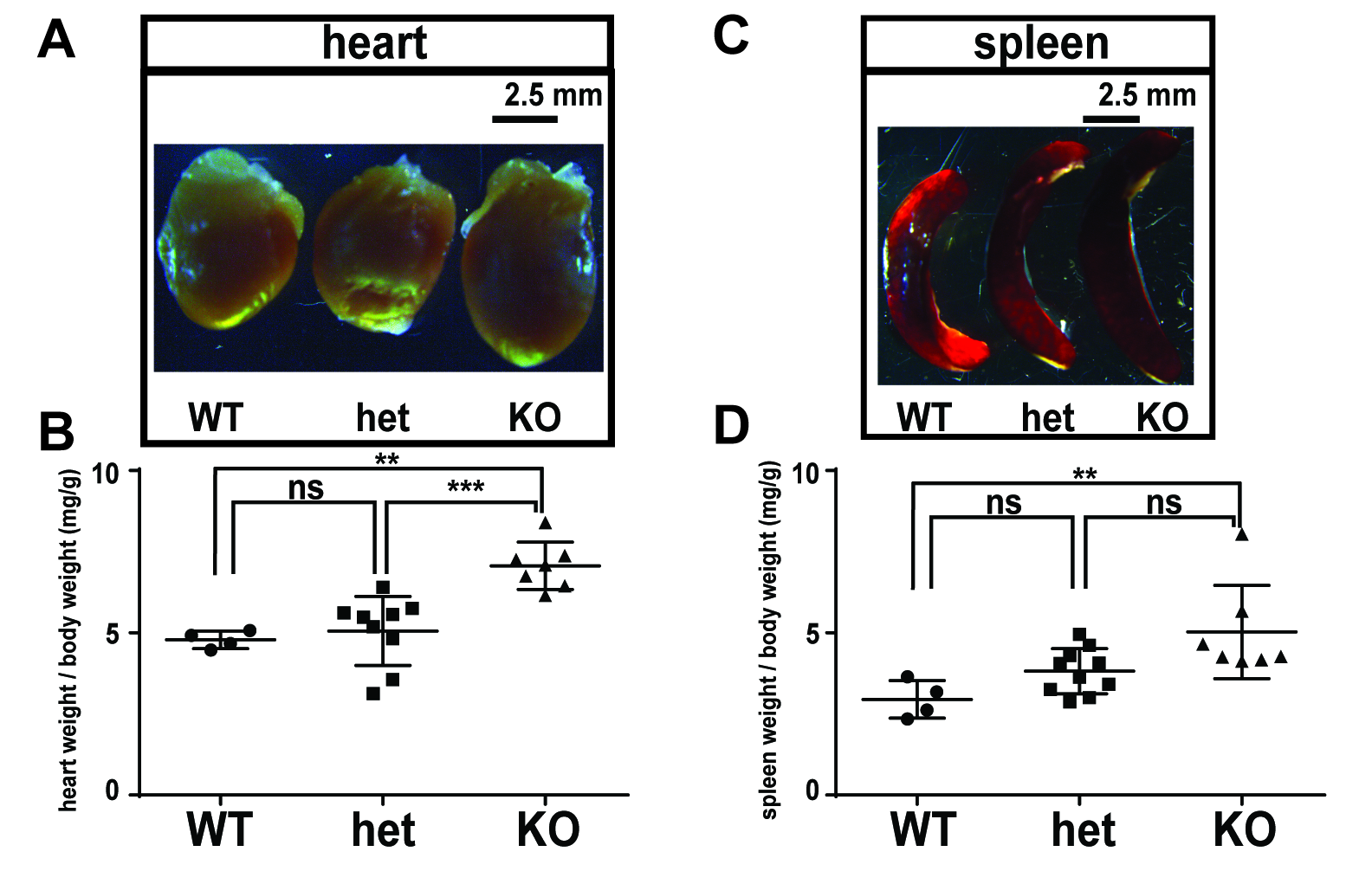

Supplement: S1 Fig — A. Bright field image of the heart from 1-month-old Gpr116 WT, heterozygous and knockout littermates. B. Weights of the heart over total body weight from 1-month-old Gpr116 WT, heterozygous and knockout littermates (n≥4 mice per genotype). C. Bright field image of the spleen from 1-month-old Gpr116 WT, heterozygous and knockout littermates. D. Weights of the spleen over total body weight from 1-month-old Gpr116 WT, heterozygous and knockout littermates (n≥4 mice per genotype). (TIF) [file pone.0137949.s001.tif]

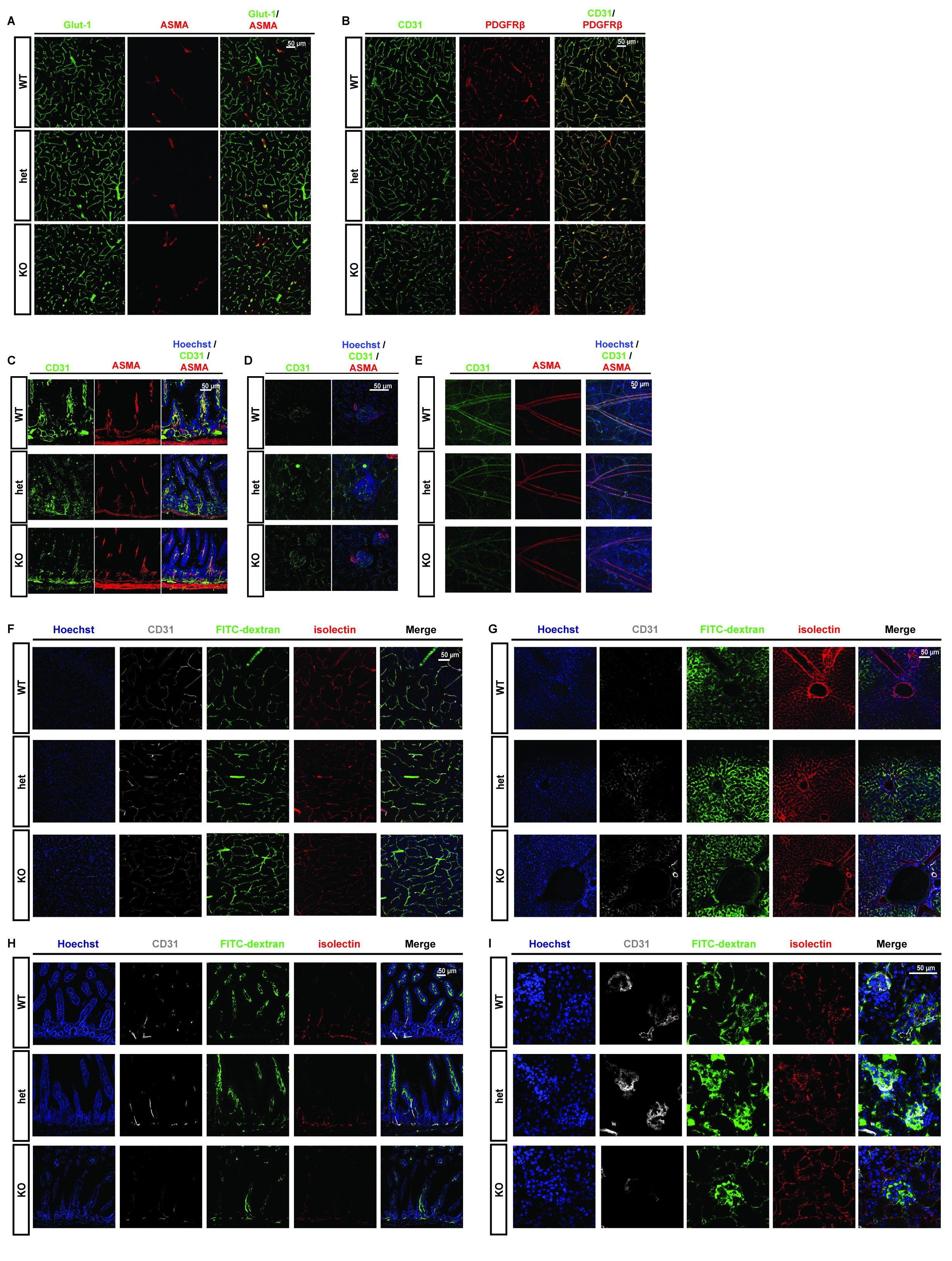

Supplement: S3 Fig — A. Vasculature of the 1-month-old brain cortex from Gpr116 WT, heterozygous and knockout littermates. Glut-1 (green) is used to visualize endothelium, and ASMA (red) to distinguish the major vessels (the images shown are representative of 2 mice per genotype). B. Vasculature of the 1-month-old brain cortex from Gpr116 WT, heterozygous and knockout littermates. CD-31 (green) is used to visualize endothelium, and PDGFRβ (red) to reveal the pericytes coverage (the images shown representative of 2 mice per genotype). C. Vasculature of the 1-month-old intestine villi from Gpr116 WT, heterozygous and knockout littermates. CD-31 (green) is used to visualize endothelium, and ASMA (red) to distinguish the major vessels (the images shown are representative of 2 mice per genotype). D. Vascular beds in 1-month-old kidney glomerulus from Gpr116 WT, heterozygous and knockout littermates. CD-31 (green) is used to visualize endothelium, and ASMA (red) to distinguish the afferent arteriole (the images shown are representative of 2 mice per genotype). E. Vascular beds in 1-month-old outer ear from Gpr116 WT, heterozygous and knockout littermates. CD-31 (green) is used to visualize endothelium, and ASMA (red) to distinguish the major vessels (the images shown are representative of 2 mice per genotype). F. Isolectin (red) and FITC-dextran (green) distribution in P21 cerebral cortex from Gpr116 WT, heterozygous and knockout littermates. CD31 (grey) is used to visualize the endothelium (the images shown are representative of 3 mice per genotype). G. Isolectin (red) and FITC-dextran (green) distribution in P21 liver from Gpr116 WT, heterozygous and knockout littermates. CD31 (grey) is used to visualize the endothelium (the images shown are representative of 3 mice per genotype). H. Isolectin (red) and FITC-dextran (green) distribution in P21 intestinal villi from Gpr116 WT, heterozygous and knockout littermates (the images shown are representative of 3 mice per genotype). I. Isolectin (re [file pone.0137949.s003.tif]

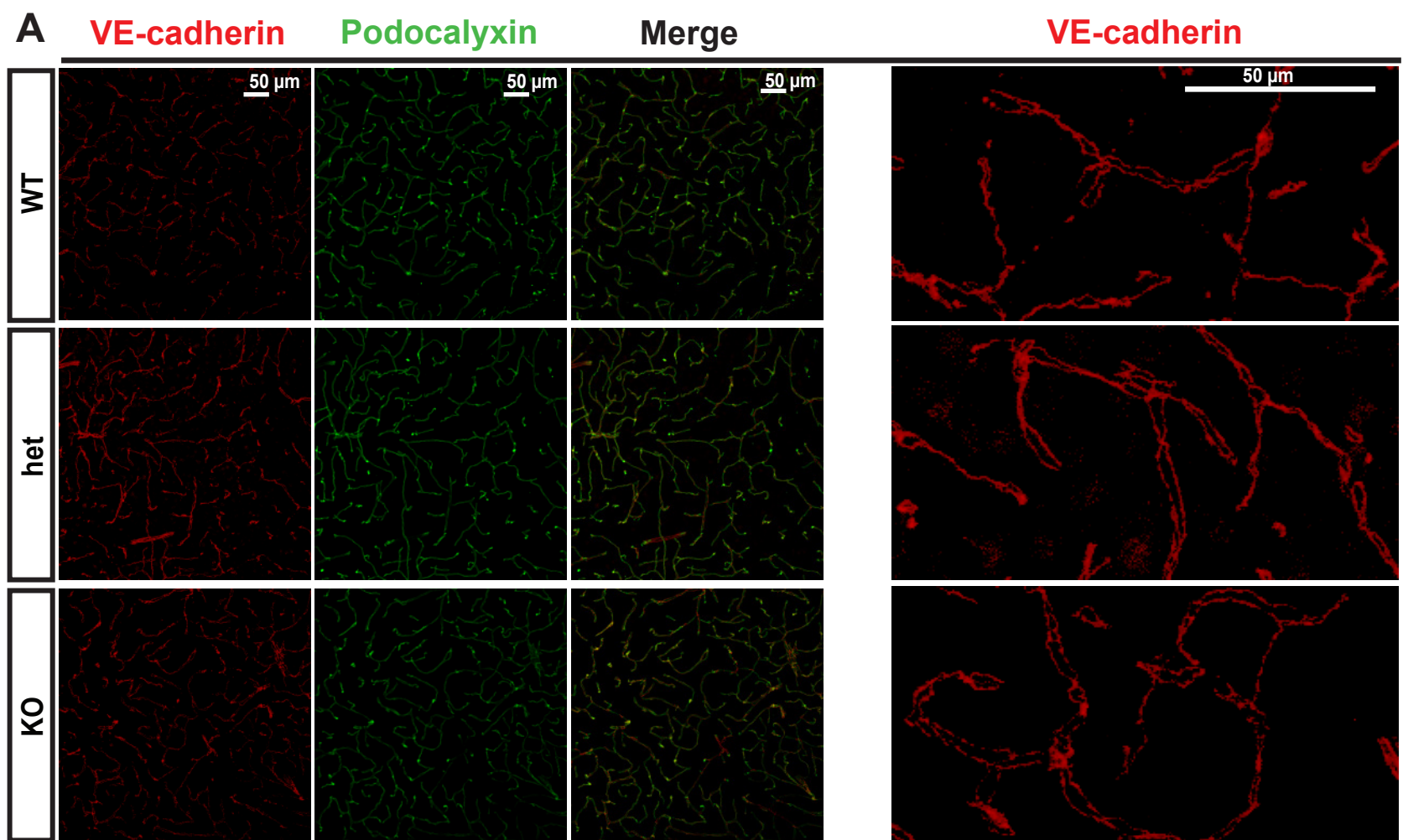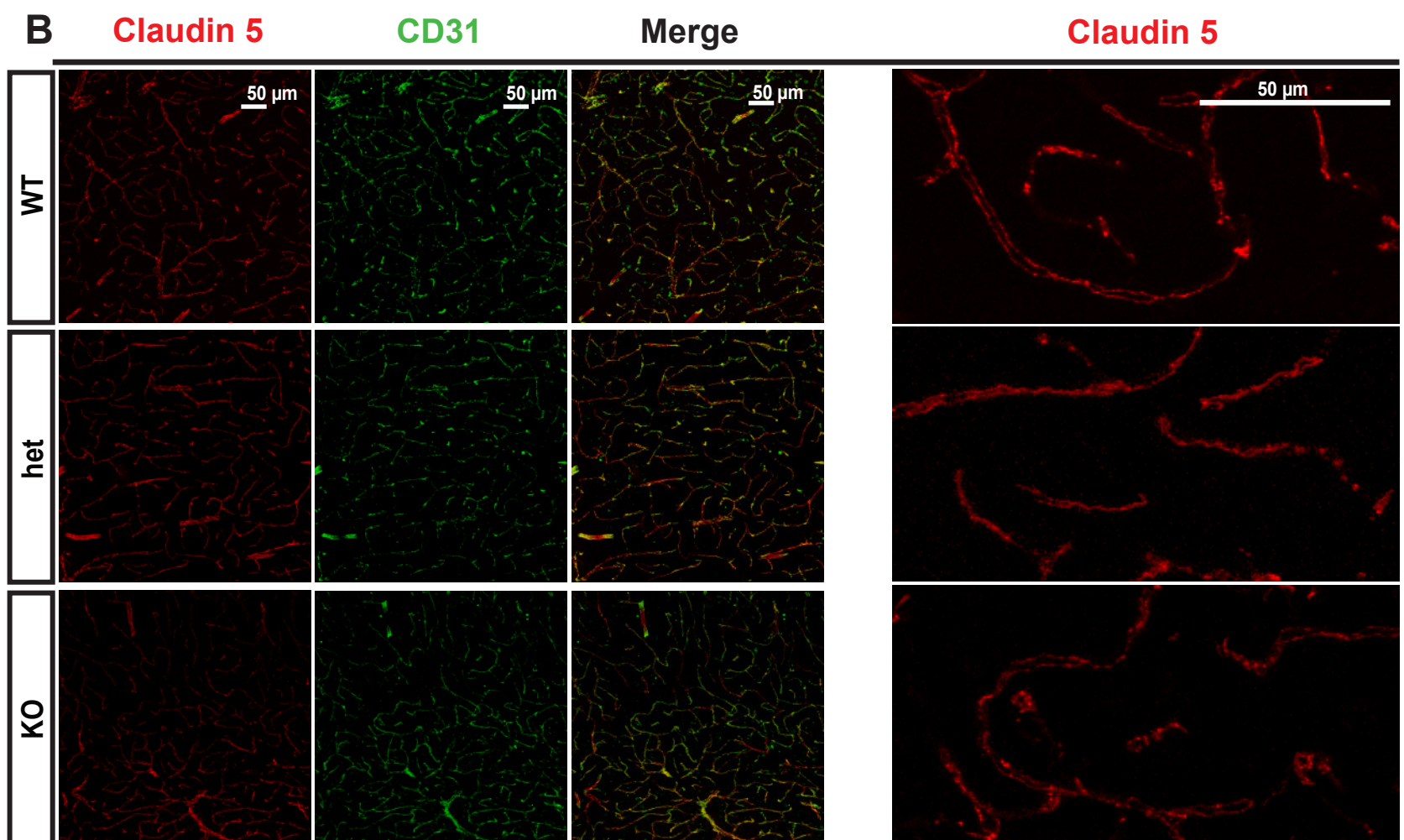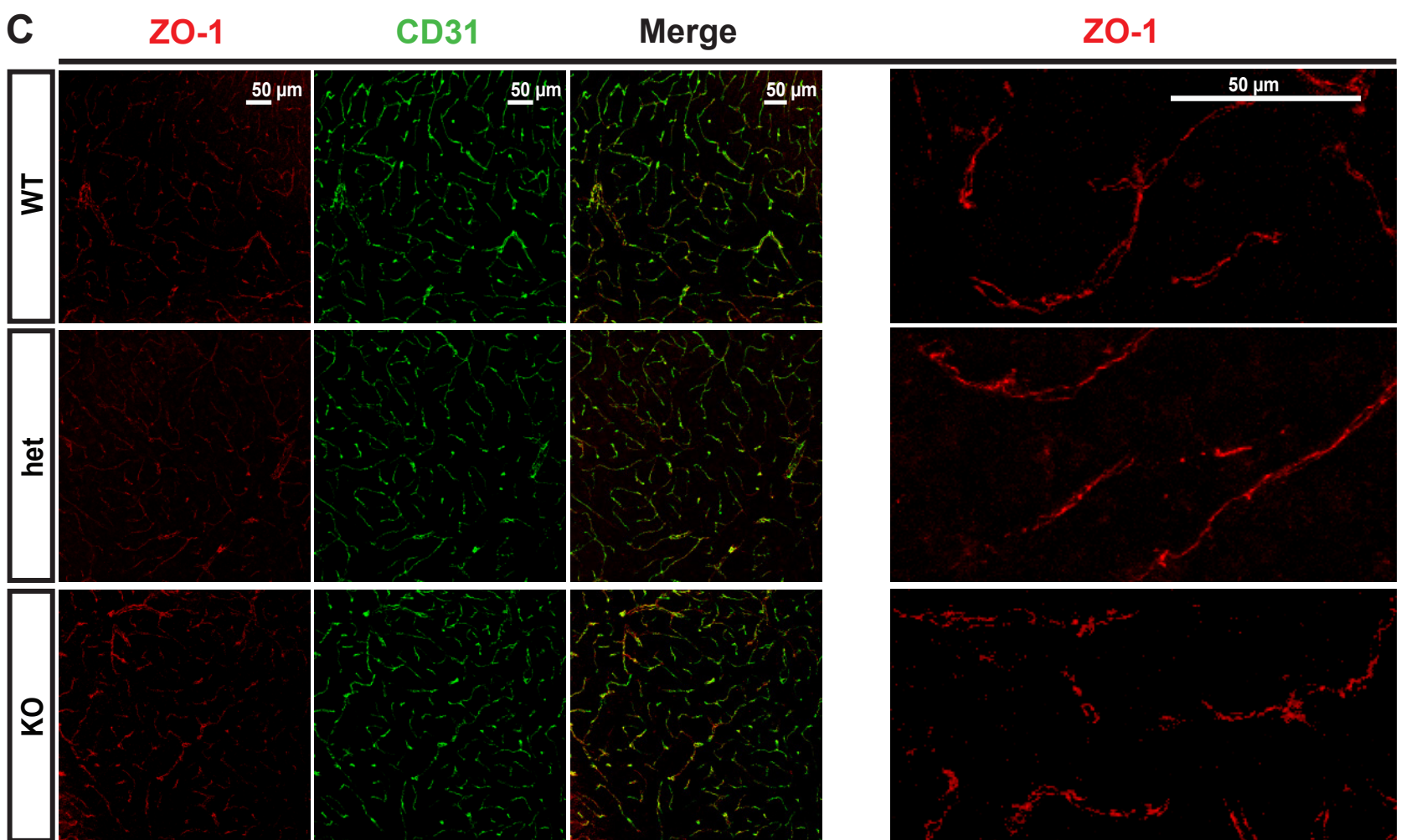

Supplement: S4 Fig — A. Adherens junction staining in 1-month-old brain cortex from Gpr116 WT, heterozygous and knockout littermates. VE-cadherin (red) is used to visualize adherens junctions, and podocalyxin (green) to delineate the vessels. A higher magnification of VE-cadherin is shown on the right side of the figure (the images shown are representative of 2 mice per genotype). B. Tight junction patterning in 1-month-old brain cortex from Gpr116 WT, heterozygous and knockout littermates. Claudin-5 (red) is used to visualize tight junctions, and CD31 (green) to delineate the vessels. A higher magnification of the claudin-5 is shown on the right side of the figure (the images shown are representative of 2 mice per genotype). C. Tight junction staining in 1-month-old brain cortex from Gpr116 WT, heterozygous and knockout littermates. Another tight junction marker, Zo-1 (red) is used and CD31 (green) to delineate the vessels. A higher magnification of the ZO-1 is shown on the right side of the figure (the images shown are representative of 2 mice per genotype). (PDF) [file pone.0137949.s004.pdf]

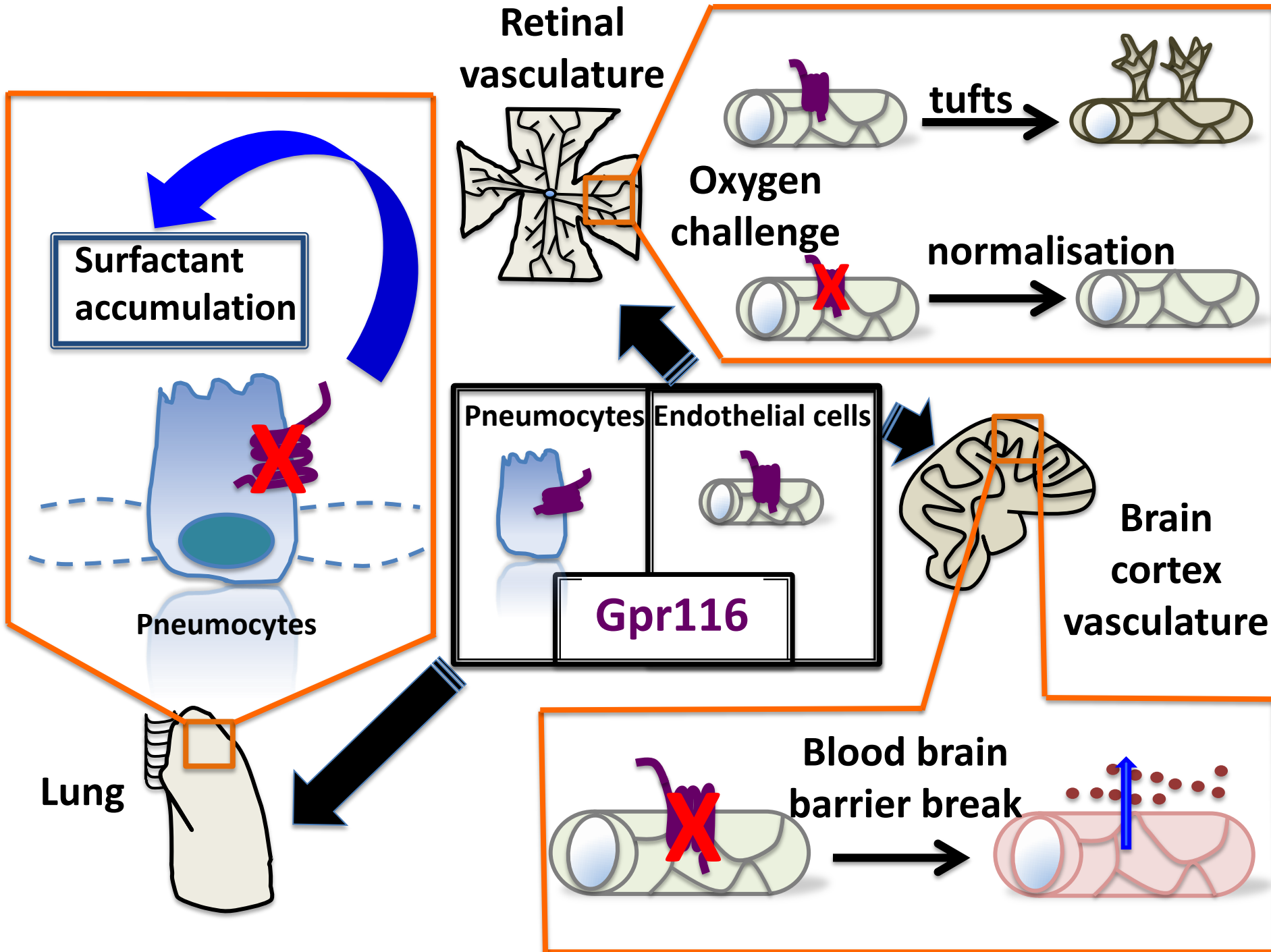

Supplement: S5 Fig — (PDF) [file pone.0137949.s005.pdf]
